# Bio‐Waste‐Derived Hard Carbon Anodes Through a Sustainable and Cost‐Effective Synthesis Process for Sodium‐Ion Batteries

**DOI:** 10.1002/cssc.202201713

**Published:** 2022-11-11

**Authors:** Hyein Moon, Alessandro Innocenti, Huiting Liu, Huang Zhang, Marcel Weil, Maider Zarrabeitia, Stefano Passerini

**Affiliations:** ^1^ Helmholtz Institute Ulm (HIU) Helmholtzstrasse 11 89081 Ulm Germany; ^2^ Karlsruhe Institute of Technology (KIT) P.O. Box 3640 76021 Karlsruhe Germany; ^3^ Institute for Technology Assessment and Systems Analysis (ITAS) Karlsruhe Institute of Technology (KIT) 76021 Karlsruhe Germany

**Keywords:** electrode materials, energy storage, hard carbon, life cycle assessment, sodium-ion battery

## Abstract

Sodium‐ion batteries (SIBs) are postulated as sustainable energy storage devices for light electromobility and stationary applications. The anode of choice in SIBs is hard carbon (HC) due to its electrochemical performance. Among different HC precursors, bio‐waste resources have attracted significant attention due to their low‐cost, abundance, and sustainability. Many bio‐waste materials have been used as HC precursors, but they often require strong acids/bases for pre‐/post‐treatment for HC development. Here, the morphology, microstructure, and electrochemical performance of HCs synthesized from hazelnut shells subjected to different pre‐treatments (i. e., no pre‐treatment, acid treatment, and water washing) were compared. The impact on the electrochemical performance of sodium‐ion cells and the cost‐effectiveness were also investigated. The results revealed that hazelnut shell‐derived HCs produced *via* simple water washing outperformed those obtained *via* other processing methods in terms of electrochemical performance and cost–ecological effectiveness of a sodium‐ion battery pack.

## Graphical abstract

**Go nuts**: Hard carbon, the anode of choice for sodium‐ion batteries, is developed using highly abundant bio‐waste precursor material in Europe (i. e., hazelnut shell) towards a sustainable and cost‐effective synthetic process, supporting the circular economy. The results reveal improved electrochemical properties, such as initial coulombic efficiency, specific capacity, long‐term stability, and rate capability.

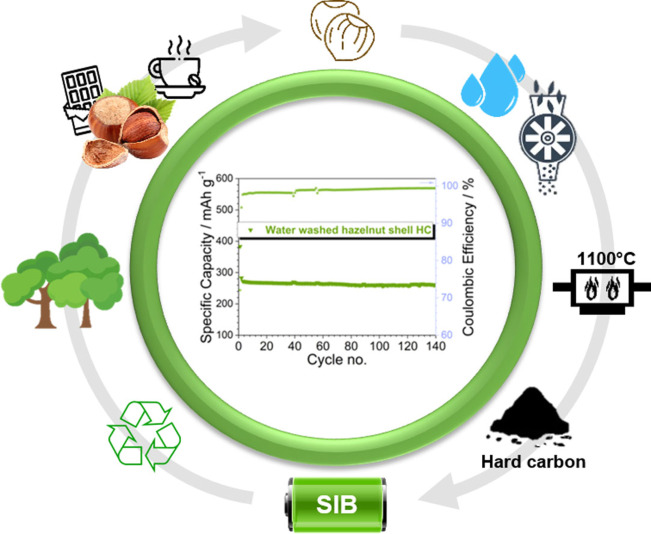

## Introduction

Sodium‐ion batteries (SIBs) are one of the most promising next‐generation energy storage devices for light electromobility and large‐scale stationary applications. Contemporary Amperex Technology Co., Ltd. (CATL) announced the manufacturing of commercial SIBs by 2023 for various transportation electrification scenarios, especially for cold areas.^[1]^ In addition, Natron Energy Inc. from the United States also announced their mass production plan, the world's largest SIB plant, at Clarios Meadowbrook facility from 2023.^[2]^ Finally, the acquisition of Faradion UK Ltd., one of the pioneers of the SIB prototype technology, by Reliance New Energy Solar Ltd. (RNESL) in 2021 implies that the commercialization of the sodium‐technology is within reach.^[3]^

The main advantages of SIBs over lithium‐ion batteries (LIBs) are their potentially lower cost and sustainability. SIBs are not dependent on critical raw materials, such as lithium, cobalt, and nickel,[^4^, ^5^] which also cause high environmental impacts.[^6^, ^7^, ^8^] As a result, many sodium‐based prototypes have been proposed. Different cathode families have been implemented, such as polyanion compounds, layered oxides, or Prussian blue analogues (PBAs). Meanwhile, most sodium‐based prototypes are based on hard carbon (HC) anode, including CATL's sodium‐cells and Faradion Ltd.,[^1^, ^9^, ^10^, ^11^, ^12^, ^13^, ^14^] which published their extensive work on developing prototype sodium‐ion cells with their proprietary HC anode.^[13]^ HCs are considered the best carbonaceous anode of choice in SIBs due to the impossibility of the graphite to insert Na^+^ ions in carbonate‐based electrolytes.^[15]^ Nevertheless, HC anodes have several disadvantages and challenges, such as low density and unstable solid electrolyte interphase (SEI) formation, which causes low initial coulombic efficiency (ICE) and power capability.[^4^, ^16^, ^17^] Therefore, optimized HC anodes with improved electrochemical properties and high density must be developed to increase the competitiveness of SIBs against the state‐of‐the‐art LIBs.

Different carbon precursors can be employed to develop HC materials. The traditional precursors (sugar, polymers, etc.) and their synthetic routes (acidic/basic pre‐/post‐treatment/s) have the disadvantages of high cost, low yield, and/or low sustainability, which ultimately reduces the viability of SIBs substantially.^[18]^ Therefore, alternative HC sources should be discovered. Among different carbon precursors, bio‐waste materials are attractive and “green” alternatives, which promote the circular economy and remarkably reduce battery costs, providing considerable breakthroughs in the renewable energy market. In fact, industrial agriculture generates 140 billion metric tons of bio‐waste every year.^[19]^ Moreover, bio‐waste is still under‐utilized and often openly burned, releasing CO_2_, volatile organic compounds, and oxides, resulting in interrupted carbon neutrality and air pollution. One sustainable solution is to reuse bio‐waste for energy applications.

Various bio‐waste materials have been studied as HC precursors. The pomelo peel‐derived HC anode delivers 181 mAh g^−1^ at 200 mA g^−1^ with low ICE of 27 %.^[20]^ The apple pomace‐derived HC anodes exhibit a capacity of 245–300 mAh g^−1^ at 20 mA g^−1^ with ICE close to 65 %, while the HC anode derived from corncob shows a specific capacity of 257 mAh g^−1^ at 20 mA g^−1^ and ICE of 57 %.[^21^, ^22^] On the other hand, the peanut shell‐ and banana peel‐derived HC anodes deliver a specific capacity from 190 to 298 mAh g^−1^ at 20 mA g^−1^ with the highest ICEs of 68 and 70 %, respectively.[^23^, ^24^, ^25^] Indeed, many other bio‐waste‐derived HC anode materials reported exhibit good electrochemical performances with excellent ICE and Na^+^ ion storage capacity.^[26]^ However, most of them suffer a poor rate capability at high current densities, as well as require high pyrolysis temperatures. In addition, it should be noted that the precursors of all aforementioned bio‐waste‐derived HCs are pre‐/post‐treated with strong acids (e. g., H_3_PO_4_, HCl) or base (e. g., KOH) to remove impurities. These treatments intrinsically prevent an industrial‐level development of such bio‐waste‐derived HCs as the manufacturing process will be prolonged and complex, with low yield, along with unsustainable acid/base waste disposal. To resolve these standing problems, it is crucial to develop bio‐waste‐derived‐HC anodes with good electrochemical performance *via* facile and sustainable synthetic methods.

Herein, a comparative investigation on the impact of the synthetic routes on the morphological, microstructural, and electrochemical properties of hazelnut shell‐derived‐HCs is reported. Hazelnut shells were selected as precursors due to their low cost ($0.15–0.25 kg^−1^).[^27^, ^28^, ^29^] Notably, hazelnut production is predominant in Turkey, with 730,000 megatons of hazelnut production, followed by Italy (160,000 megatons) in 2020/2021.^[30]^ These already large numbers can be more than doubled when considering the amount of hazelnut shells produced. In addition to the regional advantage to access the precursor, the hazelnut shells are also rich in lignin content of 48.9 %,^[31]^ which is reported to be beneficial for electrochemical performance when applied as an anode material for SIBs.^[32]^ Hence, three different synthesis routes have been carried out, such as not applying any pre‐/post‐treatment (nw‐hz‐HC), acid‐treated (at‐hz‐HC), and water‐washed (ww‐hz‐HC) HCs. In addition, the water‐washed HC was further optimized, considering industrial manufacturing, by reducing the particle size and controlling particle size distribution upon the synthetic route. The results reveal that the acid treatment is unnecessary, with the water washed hazelnut shell‐derived‐HC exhibiting an excellent specific capacity, initial coulombic efficiency, capacity retention, and rate capability. Additionally, an improved environmental benefit and cost advantages are also confirmed by losing acid treatment in the synthesis process.

## Results and Discussion

### Optimizing hard carbon performance through different synthetic routes

**Figure 1:**
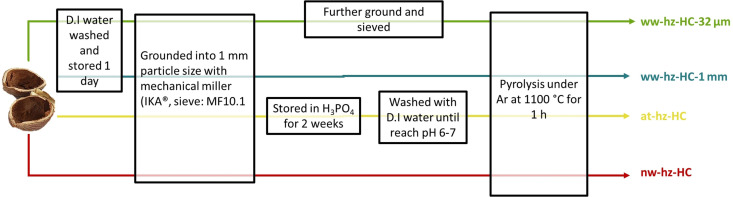
Scheme of the different synthetic routes for bio‐waste hazelnut shell‐derived HCs: not washed HC (nw‐hz‐HC), acid‐treated HC (at‐hz‐HC), water‐washed HC grounded into 1 mm particle size (ww‐hz‐HC‐1 mm) and <32 μm particle size (ww‐hz‐HC‐ 32 μm).

The bio‐waste hazelnut shell‐derived HC is developed by three synthetic routes, as schematically shown in Figure 1 (more details are provided in the Experimental Section). The main differences are that the acid‐treated HC sample (at‐hz‐HC) involved two weeks of phosphoric acid treatment following the optimized synthesis process of lignin‐based peanut shell‐derived HC.^[23]^ The phosphoric acid is used as an activating agent, which leads to the decomposition of the hemicellulose species of the hazelnut shells *via* hydrolysis.^[33]^ In addition, the phosphoric acid treatment removes unnecessary organic residues and enhances the porous structure.^[20]^ In the case of the water‐washed HC samples (ww‐hz‐HC), the hazelnut shells are only washed and stored in deionized (D.I.) water before grinding to remove the impurities, which is sustainable and scalable processing.

**Figure 2:**
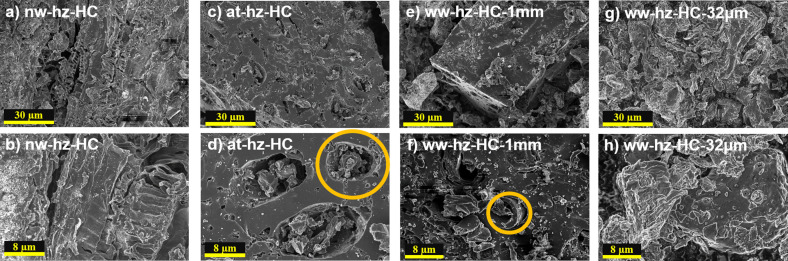
SEM images of (a,b) not washed (nw‐hz‐HC), (c,d) acid‐treated (at‐hz‐HC), and water‐washed grounded into particle size of (e,f) 1 mm (ww‐hz‐HC‐1 mm) and (g,h) 32 μm (ww‐hz‐HC‐32 μm) hazelnut shell‐derived HCs.

**Figure 3:**
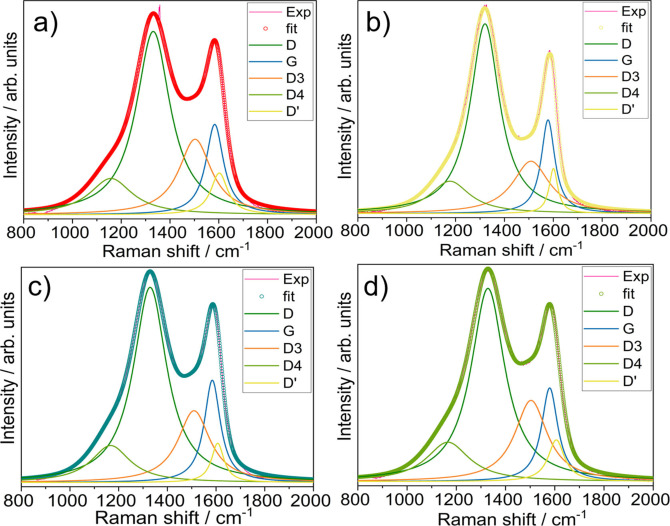
Deconvoluted Raman spectra of (a) not washed (nw‐hz‐HC), (b) acid‐treated (at‐hz‐HC), and water‐washed (c) 1 mm (ww‐hz‐HC‐1 mm) and (d) 32 μm (ww‐hz‐HC‐32 μm) hazelnut shell‐derived HCs.

The morphological and structural properties of the nw‐hz‐HC, at‐hz‐HC, and ww‐hz‐HC in both sizes have been studied by scanning electron microscope (SEM; Figure 2), energy‐dispersive X‐ray spectroscopy (EDX; Figure S1), Raman spectroscopy (Figure 3), and X‐ray diffraction (XRD; Figure S2) analyses. SEM images reveal that the synthetic route influences the morphology. The nw‐hz‐HC (Figure 2a,b) exhibits a bark‐like compact texture with an unclean surface. The surface is covered with organic and inorganic species, where the white dots in Figure 2b correspond to potassium species (confirmed by EDX, see Figure S1). In addition, a coil‐like cellulose feature can be found inside the scratched surface, which probably corresponds to carbonized pectin or hemicellulose traces.^[34]^ Meanwhile, the at‐hz‐HC (Figure 2c,d) and ww‐hz‐HC (Figure 2e–h) exhibit much cleaner surfaces (lower impurities), indicating that the acid and washing step effectively removes surface impurities of the hazelnut shells. The at‐hz‐HC shows a clean surface with more scratched and larger pores than other HC samples, suggesting that the most organic and inorganic species were washed off. A vessel‐like feature observed in ww‐hz‐HC‐1 mm (Figure 2f) is eliminated for at‐hz‐HC (Figure 2d), implying that the acidic reaction decomposes the cellulose microfibrils inside the biological cell block. Meanwhile, the biological cell block, mainly composed of lignin, remained (see Figure 2e). Lastly, comparing two water‐washed HC samples with different particle sizes, the SEM images show that the block‐like features are broken from the more intense grinding process (ww‐hz‐HC‐32 μm), leading to rounded HC particles and wrinkled surface owing to the direct influence of pyrolysis on each smaller particle.

Raman spectra (Figure 3) display the characteristic broad band of disordered carbon materials with D‐band at 1340 cm^−1^ and a sharper G‐band at 1580 cm^−1^. The D‐band represents a disorder‐induced band, corresponding to the vibrational mode of disordered or defected graphitic lattice, while the G‐band shows the vibrational mode of sp^2^ carbon atoms in the graphitic lattice. However, it should be noted that the graphitic lattice in the HC structure is far different from the conventional graphitic lattice, as it contains the lattice in the form of turbostratic layered pseudo‐graphitic nano‐domains (TLPG‐NDs).^[35]^ In addition, the Raman spectrum of disordered carbon materials is deconvoluted into three more components, such as D’‐ (1600 cm^−1^), D3‐ (≈1500 cm^−1^), and D4‐ (≈1200 cm^−1^) bands.^[36]^ The calculated relative area of each band within each sample is included in Table 1.

**Table 1:** Relative area of each band within samples in percentage, as well as D‐ and G‐band area ratio (*A*_D_/*A*_G_) and calculated crystalline size (*L*_a_) of the hazelnut shell‐derived‐HC powders in Raman spectroscopy.

Sample	D‐band [%]	G‐band [%]	D3‐band [%]	D4‐band [%]	D’‐band [%]	*A*_D_/*A*_G_	*L*_a_ [nm]
nw‐hz‐HC	49.24	13.37	19.92	11.55	5.92	3.68	10.47
at‐hz‐HC	52.88	10.68	18.46	11.62	6.36	3.18	12.12
ww‐hz‐HC‐1 mm	51.88	14.03	18.89	10.98	4.21	3.70	10.41
ww‐hz‐HC‐32 μm	49.42	12.35	20.54	12.18	5.52	4.00	9.63

Evident variations on Raman spectra are observed between samples, although the same bio‐waste precursor and pyrolysis condition is used. Comparing the D‐band concentration, the at‐hz‐HC and ww‐hz‐HC‐1 mm samples unexpectedly present a higher relative concentration of defected graphitic lattice. This suggests that the pre‐treatments may induce disorder or defects at the graphitic lattice. However, considering the following relative G‐band in both samples, the at‐hz‐HC seems to have a much lower concentration of TLPG‐NDs, while ww‐hz‐HC‐1 mm contains the most. This is confirmed by the characteristic (002) reflections at 2*θ*=22.0° in XRD (Figure S2 and Table S5), where the intensity of at‐hz‐HC is the lowest while ww‐hz‐HC‐1 mm shows the highest.

Meanwhile, nw‐hz‐HC and ww‐hz‐HC‐32 μm display the most intense D3‐band contribution, which is related to the vibrational mode of amorphous carbon, suggesting that they contain adsorbed molecules or functional groups. In addition, the ww‐hz‐HC‐32 μm, nw‐hz‐HC, and at‐hz‐HC exhibit a more pronounced D4‐band than ww‐hz‐HC‐1 mm. Considering that the D4‐band is attributed to sp^2^–sp^3^ stretching motions and surface‐level ionic impurities, the three HCs (except ww‐hz‐HC‐1 mm) are expected to contain more surface impurities and/or a high degree of sp^2^–sp^3^ species. The elemental mapping analysis performed by EDX confirms that the nw‐hz‐HC and ww‐hz‐HC‐32 μm contain more metal compounds, such as potassium and calcium (see Figure S1). The higher metal compound concentration in ww‐hz‐HC‐32 μm than ww‐hz‐HC‐1 mm suggests that the resultant metal compounds originated from the hazelnut shells’ minerals.^[31]^ On the contrary, the D4‐band contribution in at‐hz‐HC corresponds to sp^2^–sp^3^ polyene‐like carbon rather than the metal compounds, as indicated in EDX [later confirmed by X‐ray photoelectron spectroscopy (XPS)]. Lastly, the at‐hz‐HC exhibits the highest concentration of the D’‐band, indicating a high surface graphene layer concentration.

The area ratio between D‐ and G‐bands (*A*_D_/*A*_G_) is calculated to roughly estimate the HC's degree of disorder in the crystallites (see Table 1). The at‐hz‐HC shows the lowest *A*_D_/*A*_G_ ratio (3.18), followed by the nw‐hz‐HC (3.68) and ww‐hz‐HC‐1 mm (3.70), indicating that the degree of disorder is affected by the acid pre‐treatment but not significantly with water washing. The ww‐hz‐HC‐32 μm exhibits the highest degree of disorder (4.00). Furthermore, in‐plane crystallite size (*L*_a_) can be calculated following Tuinstra Koenig's equation.^[37]^ As expected, *L*_a_ increases with decreasing degree of disorder, at‐hz‐HC having the largest crystallite size (12.12 nm), followed by nw‐hz‐HC (10.47 nm) and ww‐hz‐HC (10.41 nm for 1 mm and 9.63 nm for 32 μm).

In summary, the nw‐hz‐HC and ww‐hz‐HC‐32 μm contain the highest amorphous carbon structure and a large concentration of terminal functional groups, sp^2^–sp^3^ carbons, and surface ionic impurities. In addition, the ww‐hz‐HC‐32 μm exhibits the highest degree of disorder resulting in the smallest in‐plane crystallite size. On the other hand, the at‐hz‐HC and ww‐hz‐HC‐1 mm contain a higher disorder/defected graphitic lattice, but the at‐hz‐HC consists of the lowest concentration of TLPG‐NDs, the ww‐hz‐HC‐1 mm exhibits the highest one. Nonetheless, the structures of all samples are not markedly different, resulting from the same pyrolysis temperature. This indicates that the pre‐treatment condition is less influential on the final HCs microstructure, contrary to that observed in the morphological properties.

**Figure 4:**
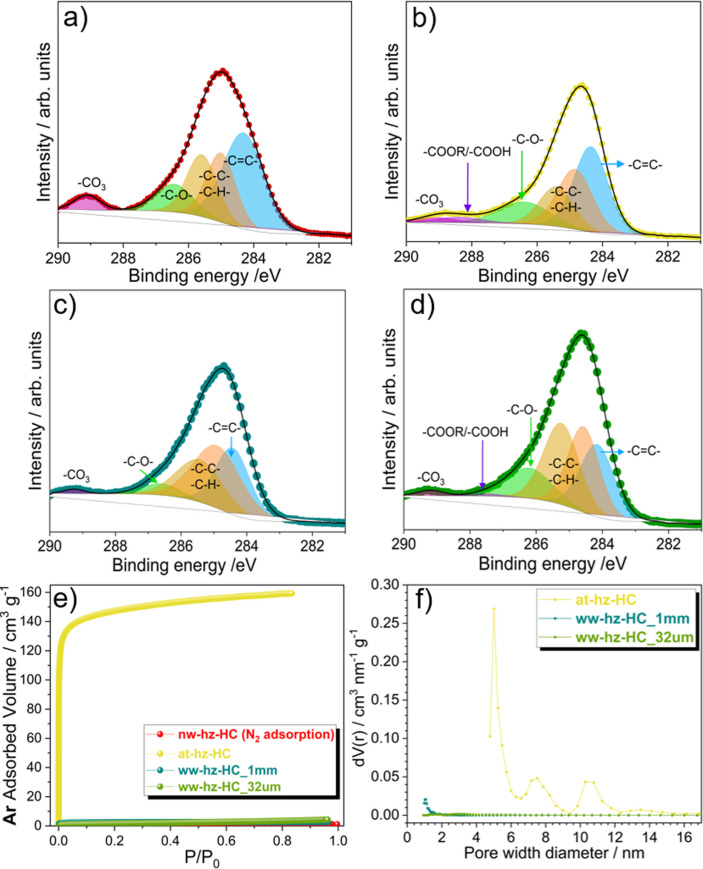
C 1s photoelectrons of (a) nw‐hz‐HC, (b) at‐hz‐HC, (c) ww‐hz‐HC‐1 mm, and (d) ww‐hz‐HC‐32 μm samples. (e) Ar adsorption and (f) corresponding pore size distribution determined by DFT calculation.

XPS has been carried out to further understand the effect of pre‐treatment on the surface chemistry of the hazelnut shell‐derived‐HCs. The C 1s region has been analyzed (Figure 4a–d), as well as the atomic and relative percentages of detected species in the C 1s region (Table 2). The atomic concentration calculated from XPS is in good accordance with the results obtained from EDX analysis in the HC samples ground into <1 mm particle size (nw‐hz‐HC, at‐hz‐HC, and ww‐hz‐HC‐1 mm). The observed differences can be attributed to XPS being limited to the surface region (max. 10 nm depth), whereas the EDX probes deeper level composition (0.5–2 μm). As expected, the at‐hz‐HC and ww‐hz‐HC‐1 mm have higher carbon content than nw‐hz‐HC. The at‐hz‐HC presents a much cleaner surface than ww‐hz‐HC‐1 mm, comparable to the EDX result. Therefore, it is confirmed that the acid pre‐treatment and water washing steps help to remove surface impurities. Meanwhile, the C 1s region provides the contribution of sp^2^ carbon (−C=C−) in each HC, which corresponds to 29.6, 32.4, 23.0, and 21.8 % for nw‐hz‐HC, at‐hz‐HC, ww‐hz‐HC‐1 mm, and ww‐hz‐HC‐32 μm, respectively. The −C=C− concentration can be correlated with the D’‐band contribution in the Raman spectra (Table 1). Unexpectedly, the ww‐hz‐HC‐32 μm shows low surface ionic impurities by XPS, contrary to EDX. Nevertheless, this can be attributed to the ionic impurities being located at a deeper level than nm‐range surface structure, which may describe the surface level impurities from D4‐band originated from the depth where EDX is detectable. The determined surface chemistry influences the surface reactivity, which will eventually affect the electrochemical performance of the HC anodes; therefore, different electrochemical properties are expected between the HCs.

**Table 2:** Atomic concentration and relative amount of detected species in the C 1s region of hazelnut shell‐derived HC powders.

HC	Element/species [at %]										
	C	−C=C−	−C−C−	−C−H_*x*_−	−C−O−	−COOR/−COOH	−CO_3_	O	K	Ca	Si
nw‐hz‐HC	70.0	29.6	15.3	14.2	8.0	–	2.9	22.3	2.1	2.2	3.4
at‐hz‐HC	87.9	32.4	23.5	14.9	12.3	2.8	1.9	12.14	–	–	–
ww‐hz‐HC‐1 mm	78.5	23.0	30.2	19.9	4.4	–	1.0	17.8	2.1	1.6	–
ww‐hz‐HC‐32 μm	85.2	21.8	23.9	25.5	12.0	0.6	1.2	12.6	0.9	1.3	–

The pore structures of the four HCs have also been studied with gas adsorption measurements. It is known that the HCs have very narrow pores or paths as well as the surface functional groups interact with N_2_ (77 K) gas. Therefore, in 2014, IUPAC suggested Ar (87 K) gas to be used as a new standard adsorbent for micro‐/mesoporosity detection.^[38]^ Therefore, Ar (87 K) and CO_2_ (273 K) adsorptions were performed for micro‐/mesoporosity and ultramicroporosity (pore diameter <0.7 nm), respectively. The Ar isotherms in Figure 4e reveal that at‐hz‐HC has a significantly higher adsorbed volume of Ar at low relative pressure, indicating a large portion of “open” porosity, resulting in a higher Ar Brunauer‐Emmett‐Teller (BET) specific surface area (SSA) of 504.9 m^2^ g^−1^. The density functional theory (DFT) pore size distribution (Figure 4f) shows the pore width of the at‐hz‐HC ranges mainly from 5 to 9 nm. In addition, the CO_2_ adsorption of at‐hz‐HC also shows micropores (<1.5 nm) and ultramicropores (0.6 nm) concentration (Figure S3), implying that at‐hz‐HC contains a high concentration of polydisperse pore sizes. In contrast, the adsorbed volume of Ar is negligible for water washed samples in both sizes (SSA_Ar,BET_=8 and 6 m^2^ g^−1^ for 1 mm and 32 μm, respectively), which suggests that the water‐washed HCs contain much less “open” porosity/microporosity. On the other hand, the pore size distribution of water‐washed samples from CO_2_ adsorption exhibits a large variance as ww‐hz‐HC‐1 mm contains a much higher concentration of ultramicropores with the size of 0.5–0.6 nm (SSA_CO2,BET_=61.7 m^2^ g^−1^). Meanwhile ww‐hz‐HC‐32 μm does not show any signs of ultramicroporosity (SSA_CO2,BET_=1.7 m^2^ g^−1^). Interestingly, nw‐hz‐HC did not show any porous structure, in agreement with the compact and closed morphology observed in SEM images (Figure 1a,b). The open porosity, as well as the surface chemistry are known to affect the ICE, meaning differences in ICE among the HC anodes are expected.

**Figure 5:**
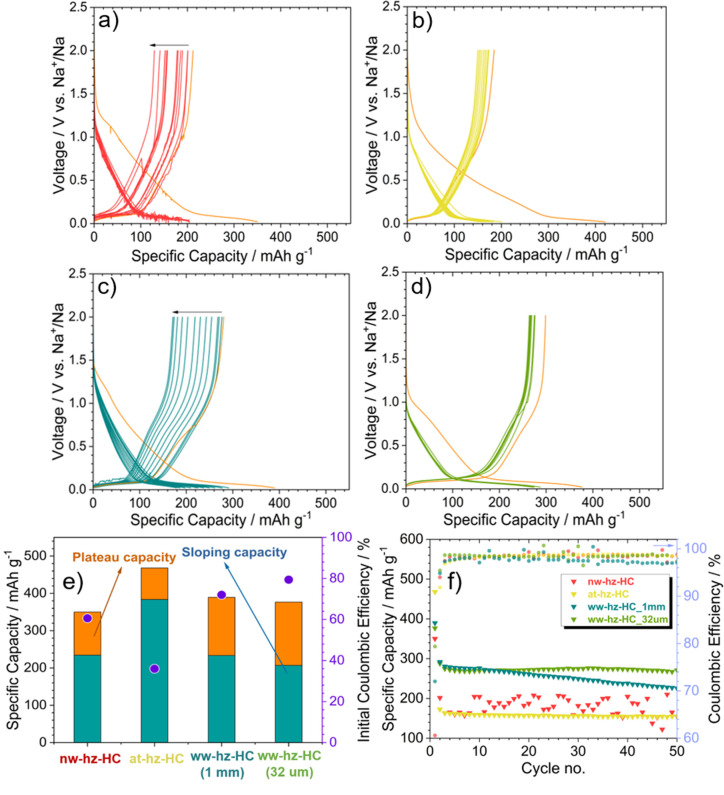
Potential profiles of (a) nw‐hz‐HC, (b) at‐hz‐HC, (c) ww‐hz‐HC‐1 mm, and (d) ww‐hz‐HC‐32 μm hazelnut shell‐derived HC anodes in half cell configuration in the voltage range of 2.0 to 0.02 V vs. Na^+^/Na. (e) Corresponding first cycle discharge capacity, the capacity contribution for two different voltage ranges and ICE (purple dots), and (f) cycling performances at first cycle at 4 mA g^−1^ (the activation cycle), followed by 20 mA g^−1^. Counter and reference electrode Na metal, electrolyte 1 m NaPF_6_ in ethylene carbonate/propylene carbonate (EC/PC) with 2 wt % fluoroethylene carbonate (FEC) solution. Active material mass loading: 2.2 mg cm^−2^. *T*=20±2 °C.

After extensively investigating the morphological, microstructural, and surface chemical characteristics of the four HCs influenced by different treatment routes, the electrochemical performances of each HC anode are studied and correlated with characteristics mentioned above. Figure 5a–d shows the (dis)charge voltage profiles upon electrochemical cycling of nw‐hz‐HC, at‐hz‐HC, ww‐hz‐HC‐1 mm, and ww‐hz‐HC‐32 μm, respectively (the first cycle highlighted with light green). The nw‐hz‐HC shows the formation of dendritic features from the first cycles, while at‐hz‐HC voltage profile implies the most stable but the lowest Na^+^ ion storage capacity. Both water washed samples show good Na^+^ ion storage capacities, but the 1 mm sample shows poorer cycling. Nevertheless, a typical voltage profile of HC anodes upon Na^+^ ion storage is observed for all HCs, showing a sloping regime in the high voltage region from 2.0 to 0.1 V (vs. Na^+^/Na) and a plateau regime in the low voltage region (<0.1 V vs. Na^+^/Na). Despite the voltage profiles being similar, the capacity contribution at the two specific voltage ranges is slightly different, which can be associated with the microstructural/morphological properties. Indeed, the d*Q*/d*V* plots (Figure S4) also reveal rather similar Na^+^ ion storage kinetics for all the HCs at low potential, exhibiting reversible (de)sodiation reaction upon cycling. Meanwhile, at high potential, some differences are observed. The reactions occurring above 1.0 V (vs. Na^+^/Na) are attributed to the Na^+^ ion storage on the defects and/or terminal group, as shown in ww‐hz‐HC‐1 mm, and ww‐hz‐HC‐32 μm.[^39^, ^40^] The 1.0–0.3 V (vs. Na^+^/Na) peaks are associated with electrolyte decomposition and SEI formation. The at‐hz‐HC shows a more pronounced peak indicating a high reactivity toward electrolyte decomposition and, in turn, SEI formation due to the high SSA detected by BET, also confirmed by the low ICE (see Figure 5e).

In Figure 5e, the first cycle capacity contributions of the two specific voltage ranges are plotted with corresponding ICE. The at‐hz‐HC sample presents the most significant contribution of the sloping potential capacity, while the others exhibit similar sloping capacity. In contrast, the water washed HCs present a higher contribution to the plateau capacity. It should be mentioned that there is still a great deal of controversy on the Na^+^ ion storage mechanism in HC anodes due to the complex structural nature of HCs.[^39^, ^40^, ^41^] Despite the controversy, according to recent studies,[^42^, ^43^] the high‐voltage sloping region is usually correlated with the Na^+^ ion adsorption at the defects and functional groups, while the low‐voltage plateau region is ascribed to the Na^+^ ion insertion into the TLPG‐NDs and/or nanopore filling.^[40]^ The nw‐hz‐HC exhibits a higher contribution of the sloping capacity than the plateau owing to the high concentration of surface impurities, as indicated by the EDX and XPS. However, the dendrite formation in nw‐hz‐HC hindered a rational correlation of the microstructural properties and cell performance. In the case of at‐hz‐HC, the sloping capacity contribution is mainly affected by its microporous structure. As previously mentioned, it is known that the large open porosity and SSA (504.9 m^2^ g^−1^) promote an excessive electrolyte decomposition in the high‐voltage region resulting in a thick SEI, as indicated by the lowest ICE of 36 % and broad band in the d*Q*/d*V* graph between 1.0 and 0.2 V (Figure S4). Therefore, only a limited amount of Na^+^ ions in the electrolyte are accessible for storage in TLPG‐NDs at low‐voltage region. This emphasizes the importance of ICE as well as the SEI properties to the overall Na^+^ ion storage capacity of HC anodes. The low‐voltage plateau capacity of at‐hz‐HC is significantly low, which can also be attributed to the lower concentration of the TLPG‐NDs as demonstrated in Raman and XRD data.

On the other hand, the water‐washed HCs deliver similar sloping capacity contribution due to the close SSA (SSA_Ar_,_BET_=8 and 6 m^2^ g^−1^ for 1 mm and 32 μm, respectively). In addition, the low‐voltage plateau region is also similar in capacity, despite the lower concentration of TLPG‐NDs in the 32 μm sample confirmed by XRD and G‐band in Raman. This indicates that ww‐hz‐HC‐32 μm contains more “closed” porosity/ultramicroporosity, contributing to plateau capacity according to the often‐mentioned Na^+^ ion storage process of nanopore filling at low voltage.[^43^, ^44^] However, the ww‐hz‐HC‐32 μm shows lower CO_2_ adsorption at low relative pressure than ww‐hz‐HC‐1 mm (Figure S3a). It should be noted that the CO_2_ adsorption detects pore width in the range of 0.35–0.7 nm, which suggests that the pore width of ww‐hz‐HC‐32 μm is narrower than 0.35 nm (see Figure S3c).[^45^, ^46^] The distinctive difference between water washed samples in the first cycle behavior is spotted in their ICEs of 72.0 and 79.4 % for ww‐hz‐HC‐1 mm and ww‐hz‐HC‐32 μm, respectively, which may be attributed to the ultramicropore size distribution of ww‐hz‐HC‐1 mm (Figure S3b). The higher concentration of “defects/disorder” observed in ww‐hz‐HC‐32 μm by Raman spectroscopy is expected to show lower reversibility. However, the obtained ICE suggests that SSA plays the main role in the reversible capacity, more than any other structural component, as shown in Figure S5. The ICE decreases with increasing SSA, which means higher reversibility in Na^+^ ion storage in the 1st cycle due to the lower degree of electrolyte decomposition. In summary, the interpretation of the first voltage profiles reveals that even though there is a slight variance in the HC microstructure, the resulting capacity contribution at different voltage regions differs due to the specific structural components in hazelnut shell‐derived‐HCs.

Furthermore, Figure 5f displays the electrochemical cycling performance of the four studied HCs. It should be noted that the cells were cycled at low current density (4 mA g^−1^) in the first cycle, as an activation cycle and homogeneous SEI formation, and in the following cycles, the current was increased to 20 mA g^−1^.[^20^, ^21^, ^22^, ^23^] One of the drawbacks of bio‐waste derived‐HC anodes is the low ICE, as indicated by the values obtained by the nw‐hz‐HC (61 %) and at‐hz‐HC (36 %). Nonetheless, in the following cycles, the at‐hz‐HC exhibits coulombic efficiencies of 98 % with good capacity retention (95 % after 50 cycles), but poor storage capacities (≈155 mAh g^−1^) probably related to the thick SEI hindering the Na^+^ ion diffusion. On the other hand, although the ww‐hz‐HC‐1 mm initially shows similar performance as ww‐hz‐HC‐32 μm, the capacity decays fast (capacity retention of 82 % after 50 cycles). The capacity fading observed in ww‐hz‐HC‐1 mm is probably attributed to the low reversibility of Na^+^ ion storage in the initial cycles. As demonstrated in Figure 5f, the coulombic efficiency of ww‐hz‐HC‐1 mm in the first 10 cycles gradually increased, while that of ww‐hz‐HC‐32 μm reached high coulombic efficiency of 98 % in the first few cycles. This indicates that in the ww‐hz‐HC‐1 mm, the electrolyte continues decomposing until forming a stable SEI, resulting in a thicker SEI and preventing the reversible Na^+^ ion storage reaction. This is evidenced by ww‐hz‐HC‐1 mm displaying unstable coulombic efficiencies between the 20th to 30th cycles. On the other hand, the ww‐hz‐HC‐32 μm exhibits the best electrochemical properties, delivering a specific capacity of 275 mAh g^−1^ with a capacity retention of 98.7 % over 50 cycles and the highest ICE, not only among all the studied HCs but also compared to those reported in the literature.[^20^, ^21^, ^22^, ^23^, ^24^, ^26^]

**Figure 6:**
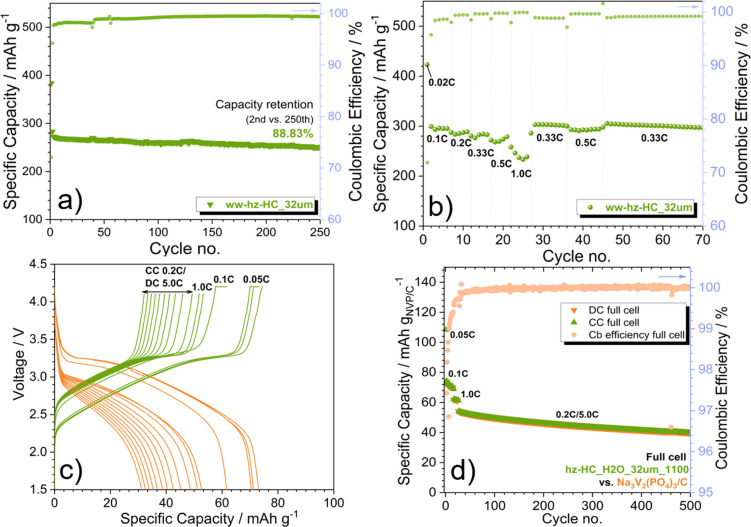
(a) Long‐term cycling and (b) C‐rate capability of ww‐hz‐HC‐32 μm in Na half cell cycled from 2.0 to 0.02 V (vs. Na^+^/Na). (c) Voltage profile and (d) C‐rate test of the full sodium‐ion cell using NVP/C as a cathode and ww‐hz‐HC‐32 μm as an anode at different current densities (1 C=120 mA g^−1^) in the voltage range of 4.2 to 1.5 V for first 30 cycles and 4.1 to 1.5 V for later ones. 1 m NaPF_6_ in PC with 2 wt % FEC as electrolyte. Active material mass loading: 8.7 mg cm^−2^ for cathode and 8.1 mg cm^−2^ for anode. *T*=20±2 °C.

Following, the long‐term cycling and rate capability of only ww‐hz‐HC‐32 μm have been investigated because of its superior electrochemical performance among other studied HCs. Figure 6a illustrates the long‐term cycling of ww‐hz‐HC‐32 μm, indicating good capacity retention of 88.83 % over 250 cycles. In addition, the ww‐hz‐HC‐32 μm also shows an excellent rate performance (Figure 6b), delivering 276, 268, and 233 mAh g^−1^ at 0.33, 0.5 and 1 C, respectively (voltage profiles at different C‐rates are included in Figure S6), as well as recovering the capacity after applying high current densities, exhibiting good reversibility. To the best of our knowledge, the ww‐hz‐HC‐32 μm exhibits superior rate capability than reported ones.^[26]^ Therefore, the electrochemical performance of ww‐hz‐HC‐32 μm clearly shows that the acid pre‐treatment on hazelnut shell‐derived HCs is not necessary and beneficial for developing a HC anode with an excellent specific capacity, ICE, capacity retention, and rate capability. Interestingly, controlling the particle size by mechanical grinding prior to pyrolysis eventually promoted a better performance. In short, hazelnut shell‐derived HC developed by a facile and environmentally sustainable synthetic route such as simply washing with water and pre‐grinding demonstrates an excellent electrochemical performance overcoming the reported electrochemical performance of bio‐waste derived‐HC anodes in terms of coulombic efficiency and/or specific capacity for long‐term cycling and/or rate capability.[^20^, ^21^, ^22^, ^23^, ^24^, ^26^]

Finally, the efficiency of ww‐hz‐HC‐32 μm as an anode electrode for Na^+^ ion storage has been tested in a full sodium‐ion cell using carbon‐coated Na_3_V_2_(PO_4_)_3_ (NVP/C) as cathode. The NVP/C is selected as cathode material due to the excellent initial coulombic efficiency (98.5 %), good rate capability (91 mAh g^−1^ at 20 C), and long‐term stability (98 % after 5000 cycles at 20 C).^[47]^ NVP/C exhibits a stable cycling performance with a well‐defined plateau at 3.4 V (vs. Na^+^/Na), with a reversible capacity of 95.6 mAh g^−1^, 99.9 % coulombic efficiency, and capacity retention of 87 % over 1000 cycles at 1 C in the voltage range of 4.2–2.5 V (vs. Na^+^/Na), as shown in Figure S7. Based on the obtained electrochemical performance of NVP/C and ww‐hz‐HC‐32 μm in half cell, a coin‐type full sodium‐ion cell is assembled. Figure 6c,d shows the electrochemical performance of ww‐hz‐HC‐32 μm||1 m NaPF_6_ in PC+2 wt % fluoroethylene carbonate (FEC)||NVP/C cell at room temperature. The full sodium‐ion cell delivers an initial capacity of 108.4 mAh g^−1^ (based on the cathode electrode weight) and an average operating voltage of 3.0 V at 0.05 C. Furthermore, the full sodium‐ion cell has been tested at a higher rate of 0.1, 1, and 0.2 C, delivering a capacity of 74.3, 62.1, and 54.0 mAh g^−1^_NVP/C_, respectively, with excellent coulombic efficiency (99.8 %) as well as a moderate capacity retention of 73.3 % after 500 cycles. Considering the average voltage and delivered capacity at 0.1 C, an energy density of 114 Wh kg^−1^_NVP/C+HC_ is obtained, demonstrating good performance of the full sodium‐ion cell with a bio‐waste‐derived HC anode (ww‐hz‐HC‐32 μm).

### Life cycle assessment

A cradle‐to‐gate life cycle assessment (LCA) is performed to evaluate the sustainability of the lab‐scale HC synthesis. The LCA method quantitatively assesses the different environmental impacts of a product, a synthetic process route, or a service according to ISO standards 14040/14044.[^48^, ^49^] The method covers the different stages of a product's life cycle, from raw material extraction (cradle) and transportation to production (gate), use, and final disposal (grave). The LCA study involves a wide range of impacts of different environmental indicators that are associated with the required energy and material. However, it should be noted that the impact of the use and the end‐of‐life phases is not considered for this study (therefore, cradle‐to‐gate framework). In addition, the modelling is conducted based on the primary data collected directly from the laboratory, therefore substantially higher environmental impacts are shown with respect to the reported industrial‐scale LCA study of HC production.^[50]^ Hence, this LCA study aims to provide a deeper understanding of the two different synthetic processes of hazelnut shell‐derived HCs (i. e., at‐hz‐HC and ww‐hz‐HC‐32 μm) and compare their environmental performance. Accordingly, the sustainability of HC synthesis can be improved by selection of a more sustainable synthesis route and exploring further optimization options.

**Figure 7:**
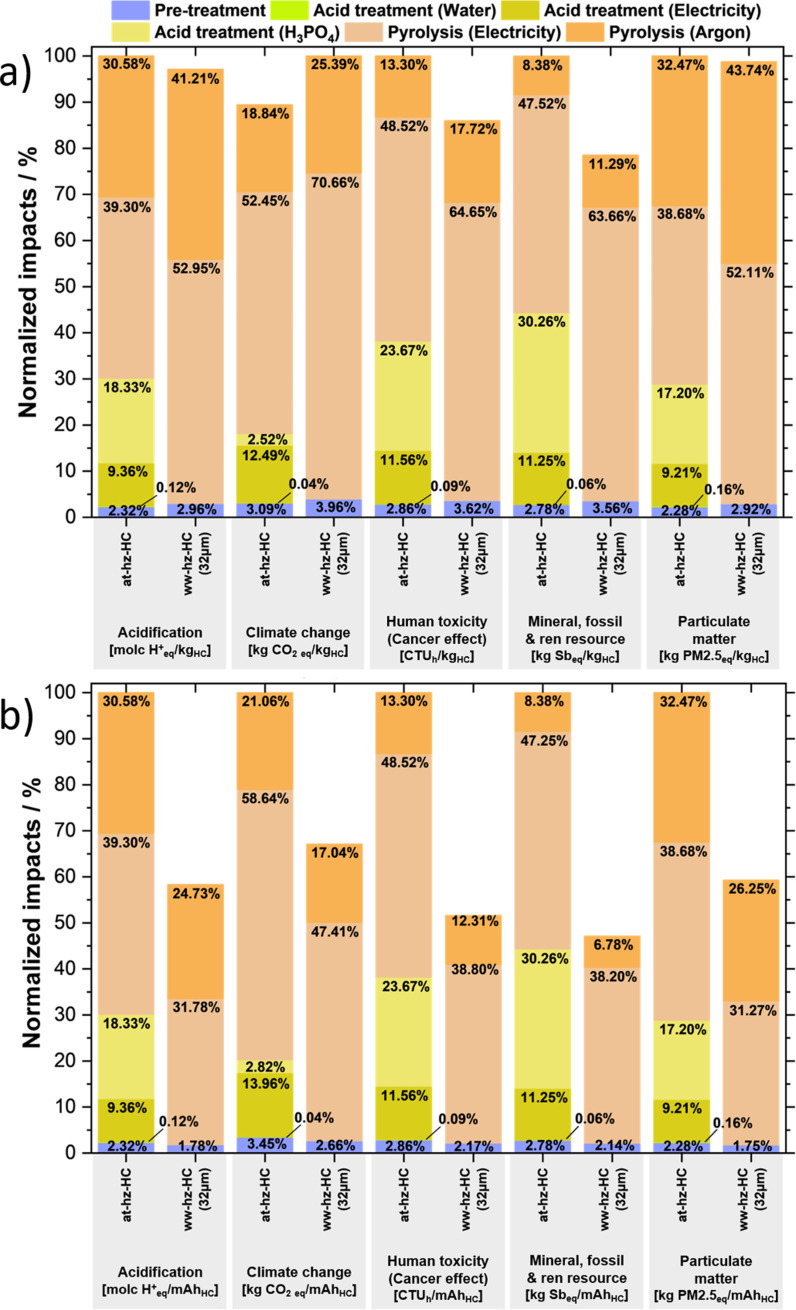
Synthesis process contribution analysis of environmental impact in two scenarios: at‐hz‐HC and ww‐hz‐HC‐32 μm, regarding functional unit of (a) 1 kg HC production and (b) 1 mAh Na^+^ storage capacity of HC anodes. The scenarios are analyzed in five different indicators, such as acidification, climate change, human toxicity, resource depletion, and particulate matter. The contribution of the synthesis process steps is shown in different colors. The percentage value is normalized by the maximum value (100 %) between two scenarios of each indicator.

The LCA results on the production of 1 kg of HC material are presented in Figure 7a (more detail in Table S6). As expected, the at‐hz‐HC presents a much higher environmental impact during the overall pre‐pyrolysis step in all considered indicators. The lowest overall contribution of the pre‐pyrolysis step for at‐hz‐HC is observed in climate change (18.14 %), which is almost five times higher than the average normalized impact of the pre‐pyrolysis step in the case of ww‐hz‐HC‐32 μm. The pre‐pyrolysis step of at‐hz‐HC is detrimental, which is shown in the four most important indicators for battery production based on the sensitivity analysis from the Product Environmental Footprint Category Rules (PEFCR) by the European commission,^[51]^ such as resource depletion (44.35 %), acidification (30.13 %), particulate matter (28.85 %), and climate change. Additionally, the cancer effect human toxicity shows the second highest contribution of the pre‐pyrolysis step (38.18 %).

Meanwhile, the most significant contribution to the synthesis process within each scenario is the electricity consumption during pyrolysis. The contribution of the overall pyrolysis step on ww‐hz‐HC‐32 μm is seemingly higher than at‐hz‐HC in all the indicators. This is due to the simpler pre‐pyrolysis steps of ww‐hz‐32 μm (78 %), which show a higher yield than at‐hz‐HC (42 %). Accordingly, the pyrolysis yield is lower for ww‐hz‐HC‐32 μm (23 %) than at‐hz‐HC (31 %). However, it should be noted that at‐hz‐HC synthesis, additional electricity is consumed for drying and ventilation, resulting in a more energy‐consuming synthesis route. Besides, due to the characteristic of cradle‐to‐gate analysis, the acid waste disposal and treatment are cut‐off in this study, which may lead to a potential underestimation of the negative impact of the acid treatment. Furthermore, the LCA performed with respect to 1 kg of HC material does not exhibit a considerable difference between the two scenarios because the analysis is focused on the production of the material rather than considering the final impact when using such material. The environmental impact of 1 mAh Na^+^ can be derived using a simplified approach by dividing 1 kg of HC to its specific capacity as shown in Figure 7b. Thus, significant deviations in two scenarios are observed considering 1 mAh Na^+^ storage capacity of final HC material in the LCA. The higher differences in the environmental impact considering the Na^+^ storage capacity is due to the higher delivered capacity of hz‐HC‐32 μm than at‐hz‐HC (2nd discharge capacity: 287.8 mAh g^−1^ for ww‐hz‐HC‐32 μm and 172.7 mAh g^−1^ for at‐hz‐HC).

Thus, in summary, from an environmental perspective the water‐washed hazelnut shells‐derived HC (ww‐hz‐HC‐32 μm) exhibits lower potential impacts and seems to be the most favorable HC for further investigation.

### Energy density and cost analysis of sodium‐ion battery packs

**Figure 8:**
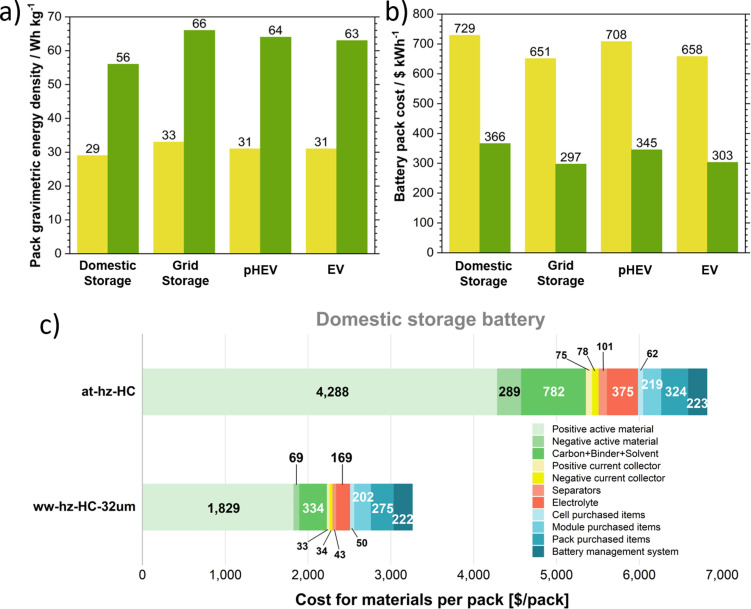
Analysis of (a) pack gravimetric energy and (b) pack cost of the four SIB configurations using as anode at‐hz‐HC (yellow) and ww‐hz‐HC‐32 μm (dark green). (c) Comparison between at‐hz‐HC and ww‐hz‐HC‐32 μm of the breakdown of the material costs for the domestic energy storage battery case.

Figure 8a,b depicts the pack gravimetric energy and the pack cost, respectively, and Figure S8 shows the volumetric energy for each of the four sodium‐ion battery configurations using at‐hz‐HC or ww‐hz‐HC‐32 μm. The sodium‐ion battery pack with an at‐hz‐HC anode has a lower specific energy, both in gravimetric and volumetric (29–33 Wh kg^−1^ and 49–61 Wh L^−1^), and higher cost ($651–729 k^−1^ Wh^−1^) than the ww‐hz‐HC‐32 μm (56–66 Wh kg^−1^, 89–120 Wh L^−1^, and $297–366 k^−1^ Wh^−1^) for all the battery packs. The main reasons for the overall inferior cost‐performance of the at‐hz‐HC are (i) low initial reversibility (low ICE), (ii) low specific capacity, (iii) the use of acid increasing the materials cost, and (iv) low average voltage of the sodium‐ion cells due to the small contribution of the HC low voltage plateau. Firstly, the ICE of at‐hz‐HC is less than half of ww‐hz‐HC‐32 μm (33.69 and 70.50 %, respectively). Note that the ICEs in cost analysis are calculated with the capacity reached at the first stable cycle. The greater SSA of the at‐hz‐HC enhances the electrolyte decomposition reaction and SEI formation, hence, the consumption of Na^+^ ions. Therefore, in a full cell configuration, the cathode must be oversized for full sodiation of the at‐hz‐HC and compensate the capacity lost.[^52^, ^53^] Considering the cost of the materials per pack in the domestic energy storage battery (Figure 8c and simulated results in Table S7) as an example, the cathode active material cost in the at‐hz‐HC configuration is 2.34 times higher ($4288 pack^−1^) than when ww‐hz‐HC‐32 μm is used as anode ($1829 pack^−1^). Consequently, the conductive carbon, binder, and solvent cost for the cathode will increase as well as the costs related to the current collectors, the separator, and the electrolyte, since the area and the size of the battery must be greater to accommodate the additional material. Figure S9 illustrates the material costs for the other three SIB systems, such as grid storage, plug‐in hybrid electric vehicle battery (pHEV), and electric vehicle (EV).

Secondly, the specific capacity of the at‐hz‐HC is 158 mAh g^−1^, lower than ww‐hz‐HC‐32 μm (265 mAh g^−1^). The lower specific capacity of the former determines an increased mass of HC to reach the required battery capacity, with a direct effect on the cost for the anode active material ($289 pack^−1^ for at‐hz‐HC vs. $69 pack^−1^ for ww‐hz‐HC‐32 μm). Thirdly, the use of phosphoric acid as chemical activation on the at‐hz‐HC anode increases the final cost of the mentioned HC. This together with the overall lower yield of the production process involving the acid treatment, makes the estimated cost of at‐hz‐HC equal to $7.60 kg^−1^, while the cost of the ww‐hz‐HC‐32 μm is $3.40 kg^−1^. Lastly, the Na^+^ ion storage mechanism in HCs is characterized by a sloping region at a low state of charge and a plateau region at a high state of charge. The larger low‐voltage plateau region is beneficial for a full cell configuration because it results in a higher average voltage. In turn, the number of cells required to satisfy the energy and power requirements of the battery pack is reduced, with consequent savings on the pack cost. In this context, the at‐hz‐HC only exhibits 32 % of the voltage profile characterized by a plateau, and the average potential of the sloping region is 0.72 V. While the ww‐hz‐HC‐32 μm HC is characterized with an extended plateau potential by 61 % of the whole capacity and 0.66 V as the average potential of the sloping region. Hence, it can be highlighted that the optimization of the four parameters (i. e., ICE, specific charge capacity, material cost, and average voltage) is crucial for developing cost‐effective HC materials for commercial SIBs.

## Conclusions

The physicochemical and electrochemical performance, and life cycle assessment (LCA) of hazelnut shell‐derived hard carbon (HC) anodes developed through four different synthetic routes has been investigated (i. e., not washed, acid‐treated, and water‐washed with two particle sizes). Also, ecological and cost analysis of sodium‐ion battery (SIB) packs has been carried out using acid‐treated or water‐washed with 32 μm particle size HC as anode and Na_3_V_2_(PO_4_)_3_ (NVP)/C as a cathode.

The pre‐treatment processing greatly influences the morphology, surface chemistry, and porosity nature of the HCs. Meanwhile, the microstructure is unaffected because the same pyrolysis temperature is applied for all the studied HCs. The degree of disorder is only affected by strong pre‐treatment, such as phosphoric acid. On the other hand, the extra grinding step to reduce the hazelnut shells into micrometer size modifies the particle morphology, as well as the surface chemistry revealing more metal compounds originating from minerals in hazelnut shells. Therefore, the chemical structure of the four studied HCs is similar; however, the differences in morphology, particle size, surface chemistry, and porosity result in distinct electrochemical behavior. A direct correlation between specific surface area (SSA) and initial coulombic efficiency (ICE) is observed, indicating that HCs with low SSA should be developed to enhance the ICE values.

Among the studied HC anode materials, the best performance is presented by the HC developed by water washing, which is a sustainable, industry‐applicable, and lower‐cost synthetic process, also supported by the LCA investigations. The water‐washed HC with 32 μm particle size delivers a specific capacity of 275 mAh g^−1^, with a capacity retention of 98.7 and 88.8 % after 50 and 250 cycles, respectively, an excellent ICE (79.4 %) and rate capability (233 mAh g^−1^ at 1 C) compared to reported bio‐waste‐derived HC anodes. Additionally, low pack cost in the four SIB packs is obtained using the water‐washed HC (w‐hz‐HC‐32 μm), mainly related to the superior electrochemical properties and the higher low‐voltage plateau contribution. This investigation provides a framework for developing well‐performing bio‐waste‐derived HC anodes for Na^+^ ion storage *via* a more sustainable synthetic, practical, and cost‐competitive synthetic process, in line with SIB philosophy, low‐cost, and sustainability.

## Experimental Section

### Materials preparation

The hazelnut shells were used as not treated and treated with two different solvents. Firstly, for the not washed HC (nw‐hz‐HC), the hazelnut shells were directly grounded into <1 mm particle size with a mechanical miller (IKA®, sieve: MF10.1) before pyrolysis to compare with other treated samples. The hazelnut shells of the acid‐treated HC sample (at‐hz‐HC) were firstly ground into <1 mm particles (IKA®, sieve: MF10.1) and stored in phosphoric acid for 2 weeks. Later, the hazelnut shells were washed with D.I. water until reaching pH 6–7, dried at 80 °C overnight, and pyrolyzed (for more detail, see Ref. [24]). Lastly, the water‐washed HC samples (ww‐hz‐HC) were synthesized by washing the hazelnut shells with D.I. water several times and storing in water for 1 day before grounding. Afterward, the shells were dried at 80 °C in oven overnight. The dried shells were then grounded into <1 mm particles for ww‐hz‐HC‐1 mm. At the same time, for the ww‐hz‐HC‐32 μm sample, the dried shells were further grounded with 0.025 mm sieve size (IKA® miller) and micrometer‐size sieving with a sieve shaker (Retsch, AS 200) until obtaining <32 μm size hazelnut shell fine powders. Each of the obtained samples with different synthetic methods was then pyrolyzed in a tubular furnace (Nabertherm, P330) at 1100 °C for 1 h under an Ar environment at the heating rate of 1 °C min^−1^. Finally, obtained HCs were hand‐ground and stored in a dry‐room (dew point <−70 °C).

### Materials characterization

The HCs’ morphologies and particle sizes were examined by a high‐resolution SEM (FE‐SEM, ZEISS) with 3 kV acceleration voltage. EDX (Aztec, Oxford Instruments) with 10 kV acceleration voltage was applied for elemental analysis.

The microstructural properties were investigated by powder XRD and Raman spectroscopy. The XRD data were recorded on a Bruker Advance D8 diffractometer with Cu radiation (K_α1,2_ *λ*=1.5406 Å, 1.5444 Å) in the 2*θ* range of 10–80°. Raman spectra were collected from a confocal InVia Raman microspectrometer (Renishaw) with a 633 nm red laser and a 50× objective lens in a back‐scattering configuration. The spectrum of each HC was collected in the range of 700–2000 cm^−1^ (5 accumulations of 10 s), and each spectrum was processed with background removal and normalization by using Labspec5 software. The processed spectrum was further deconvoluted by Gaussian‐Lorentzian function on the same software.

The surface chemistry of hazelnut shell‐derived‐HCs was evaluated by means of XPS (SPECS), equipped with a monochromatic Al X‐ray source (Al K_α_, *hν*=1486.6 eV) and PHOIBOS 150 hemispherical energy analyzer with 2D DLD detector. The C 1s spectra were calibrated using graphitic peak (−C=C−, sp^2^) at 284.4 eV as a reference. The calibrated spectra were fitted using a nonlinear Shirley‐type background and a 70 : 30 Gaussian: Lorentzian profile function with CasaXPS software.^[54]^

The porosity and SSA of the four investigated HCs were analyzed by Ar and CO_2_ adsorptions at 87 and 273 K, respectively (Quantachrome, Autosorb‐iQ‐MP/XR analyzer). Prior to Ar and CO_2_ adsorption, the HCs were degassed at 200 °C for 20 h. The pore size distribution (PSD) was calculated by the DFT method considering Ar 87 K on Carbon Slit Pore model and CO_2_ 273 K on Carbon NLDFT model, while the SSA was determined by the multipoint BET method.

### Electrochemical measurements

Electrodes were prepared by mixing 80 wt % of hazelnut shell‐derived‐HCs, 10 wt % of conductive carbon (Super C45, IMERYS), 5 or 4 wt % of carboxymethylcellulose sodium salt binder (CMC, Sigma‐Aldrich), and 5 or 6 wt % of styrene‐butadiene rubber (SBR, ZEON) for nw‐hz‐HC, at‐hz‐HC (CMC/SBR=5 : 5), and ww‐hz‐HC (CMC/SBR=4 : 6 for both particle sizes). The CMC was dissolved in D.I. water. The slurries were carried out using ball‐milling for 2.7 h (15 min milling and 5 min resting for 7 repetitions; speed of the main disk: 400 rpm; speed of the rotating plates: −800 rpm). The electrode processing of ww‐hz‐HC‐32 μm was modified to be industry‐applicable. The electrode was prepared by a planetary mixer (THINKY, ARE250) at a speed of 2000 rpm. The mixtures were cast on battery‐grade aluminum foil with wet thickness of 150 μm and dried at 80 °C overnight. 12 mm disk electrodes were punched and dried under vacuum in a glass oven at 120 °C for 20 h. Electrochemical tests were carried out in three‐electrodes Swagelok® T‐type cells. The T‐type cells were assembled in an Ar‐filled glovebox (MBraun, H_2_O and O_2_ <0.1 ppm), using sodium metal (99.8 %, Across Organics) as counter and reference electrodes, and hz‐HCs as working electrode. 1 m sodium hexafluorophosphate (NaPF_6_ battery grade, Fluorochem) dissolved in 1 : 1 wt % ethylene carbonate (EC; battery grade, UBE)/propylene carbonate (PC; battery grade, UBE) with 2 wt % of FEC (battery grade, UBE) additive was used as electrolyte, with a total volume of 240 μL electrolyte solution was soaked in glass fiber separator (Whatman, GF/D). Galvanostatic cycling and rate capability experiments were carried out in a battery tester (MACCOR, Series 4000) in the 0.02–2.0 V vs. Na^+^/Na voltage window at 20±1 °C. A specific current of 200 mA g^−1^ is defined as 1 C. The full cell was assembled using ww‐hz‐HC‐32 μm as anode and in‐house synthesized NVP/C as a cathode.^[47]^ The full cell was cycled in the 4.2–1.5 V voltage range at 0.05, 0.1, 1, and 0.2 C with respect to the cathode mass. The P/N weight ratio was 1.07.

### Life cycle assessment

Environmental impacts on producing hazelnut shell‐derived‐HC materials are analyzed using two scenarios (at‐hz‐HC and ww‐hz‐HC‐32 μm) applying a cradle‐to‐gate LCA. For the modelling, 1 kg HC or 1 mAh of Na^+^ ion storage capacity has been chosen as functional units. The life cycle impact assessment method ILCD 2011 midpoint+ is used to provide 16 different impact indicators for the two scenarios.^[55]^ The table of input and output inventories for the two scenarios are included in the Supporting Information (see Table S1). The most relevant impact indicators such as, “acidification”, “climate change”, “resource depletion”, and “particulate matter” (representatives of the respiratory inorganics) are chosen according to the product environmental footprint category rules (PEFCR) for rechargeable batteries from the European Commission.^[51]^ Additionally, “Human toxicity” is also included as the result reflects a significant difference in the two scenarios and often discussed in battery LCA studies.[^6^, ^56^] The detailed descriptions of each indicator and its unit are provided in the Supporting Information. The modelling of foreground system is based on primary data that are measured directly in the laboratory during the HC synthesis, covering the energy consumption as well as the material inputs and outputs. The energy consumption is associated with the maximum capacity of the corresponding applied devices. Note that the energy consumption of HC from lab‐scale synthesis is significantly higher compared to industrial‐scale production.^[50]^ The background system for supply chain of materials and energy is modelled from the commercial database Ecoinvent v3.8.^[57]^ Furthermore, the supply chain of the hazelnut shell precursor is considered as waste and free of environmental burden, because the focus of this study is in comparing different synthetic process routes. All the environmental impacts caused by the production process are allocated to the main product HC. Hazelnut shell residues created during the grinding process are considered as waste. The analysis is performed by using openLCA V1.11.1 software.

### Cost‐performance analysis

The Battery Performance and Cost (BatPaC) 5.0 model^[58]^ was used for comparison of the cost and energy density of different SIB packs [a small domestic energy storage system, a grid‐scale energy storage system, a plug‐in hybrid electric vehicle battery (pHEV), and a high‐end full electric vehicle (EV) battery] using acid‐treated (at‐hz‐HC) and waster‐washed (ww‐hz‐HC‐32 μm) HCs as anode, and NVP/C as cathode. The model simulates battery packs of determined rated power and capacity, considering the materials cost (active materials, conductive carbon, binder, separator, electrolyte, current collectors), the cost of the cell, module, and pack hardware (casings, pack current collectors, cooling system), the cost of labor, the investment costs for the production site, and other overheads (for more details see Ref. [58]). The input data required by BatPac software are provided in the Supporting Information (see Tables S2–S4). All the other unspecified data have been kept at the default value found in BatPac version 5.0.

## Conflict of interest

The authors declare no conflict of interest.

1

## Supporting information

As a service to our authors and readers, this journal provides supporting information supplied by the authors. Such materials are peer reviewed and may be re‐organized for online delivery, but are not copy‐edited or typeset. Technical support issues arising from supporting information (other than missing files) should be addressed to the authors.

Supporting Information

## Data Availability

The data that support the findings of this study are available on request from the corresponding author. The data are not publicly available due to privacy or ethical restrictions.
