# Supplementary material for: Bio‐Waste‐Derived Hard Carbon Anodes Through a Sustainable and Cost‐Effective Synthesis Process for Sodium‐Ion Batteries
Source: ChemSusChem. 2022 Nov 11;16(1):e202201713. doi: 10.1002/cssc.202201713 (PMC10099231; doi:10.1002/cssc.202201713)
Supplement: Supplementary file 1 — Supporting Information [file CSSC-16-0-s001.pdf]

ChemSusChem

Supporting Information

Bio-Waste-Derived Hard Carbon Anodes Through a Sustainable and Cost-Effective Synthesis Process for Sodium-Ion Batteries

Hyein Moon, Alessandro Innocenti, Huiting Liu, Huang Zhang, Marcel Weil,
Maider Zarrabeitia,* and Stefano Passerini*© 2022 The Authors. ChemSusChem published by Wiley-VCH GmbH. This is an open access article under the terms of the Creative Commons Attribution License, which permits use, distribution and reproduction in any medium, provided the original work is properly cited.

Experimental part: Life cycle assessment inventory table

Table S1. Life cycle inventory table of two scenarios of synthesis route.

	Flow	at-hz-HC	ww-hz-HC-32µm
**Input**	Hazelnut shells / kg	20.26675972	24.81824714
	Electricity, low voltage / kWh	4749.61758	5209.490466
	Water, deionised / kg	3167.72	49.63649427
	Argon / kg	506.7775382	682.7418287
	Tap water / kg	572.0455064	.
	Phosphoric acid, in 85% solution state / kg	71.69637014	.
**Output**	Hazelnut shell-derived HC powder / kg	1	1
	Waste water, average / L	32.45316234	39.74145914
	Water, vaporized / kg	36.22178733	11.03481313
	Hazelnut shell residual / kg	0.26782523	19.31132628
	Argon, emission / kg	506.778	682.742
	Waste water, acid polluted / kg	3759.539891	.

Experimental part: Cost-performance analysis

BatPac 5.0 model is used for cost analysis and energy density analysis.[1] The data required by BatPac for the active materials given as input are specified in this Supporting Information (Table S2-S4). All the other unspecified data have been kept at the default value found in the BatPac material definition sheets of version 5.0.

The assumptions made for the material parameters used in the model are:

- The $Na_3V_2(PO_4)_3$ cathode material cost was assumed to be 25 \$ kg^{-1}, and 3.2 g cm^{-3} density. The voltage profile and specific capacity correspond to internal experimental cycling data. The weight ratio between active material, a conductive additive, and a binder of the cathode electrode used in the simulations is 91:4:5.[2]

- The cost of the hard carbons (HCs) has been estimated through the sum between a fixed cost due to the production process and the raw material price considering the total yield of the final product. The fixed costs of the production processes have been estimated considering techno-economics studies of activated carbon production from bio-sources, which can be considered applicable also for HC production.[3-5] In general, these studies estimate a fixed production cost without raw material cost in the range of \$1.50-2.50 kg^{-1}, according to the different yields of the precursors and if physical or chemical activation is considered. Actualizing these results to the current date by adjusting for inflation and considering the yields measured in the laboratory of the acid treated and the water washed hazelnut shell-derived HCs, in the range of \$2.70-3.30 kg^{-1} and \$2.25-2.75 kg^{-1} of fixed production costs were assumed for the two HCs, respectively. The raw materials costs, *i.e.*, hazelnut shells and 85% phosphoric acid, were assumed to be in the range of \$0.15-0.25 kg^{-1} [6-8] and \$0.75-1.75 kg^{-1} [9] respectively, by consulting relevant sources. The amount of phosphoric acid used experimentally in the chemical activation of the acid treated HC (at-hz-HC) was adapted to an industrial process according to a relevant reference to calculate the cost of this HC.[5] In particular, it was assumed 1 L of 30% phosphoric acid for every kg of precursor.

- The weight ratio between active material, conductive additive, and binder of the anode electrode used in the simulations is 98:0:2. The N/P ratio after formation is set to 1. The initial Coulombic efficiency of the two studied HCs was estimated from experimental half-cell cycling data by dividing the stable charge capacity by the first discharge capacity. The effect of the initial Coulombic efficiency was simulated by multiplying the specific capacity of the cathode by the initial Coulombic efficiency and using the result as input for the cathode capacity in the model. The density of the electrodes (active material + binder + porosity) was assumed to be 0.900 g cm^{-3} for the at-hz-HC and be 0.950 g cm^{-3} for the ww-hz-HC-32 μm.

The input data of the HC anode and NVP cathode is presented in Table S2-S3.

Table S2. Input data of HC anode materials (at-hz-HC and ww-hz-HC-32 µm) for cost – performance analysis.

**Code**	**at-hz-HC**	**ww-hz-HC-32 µm**
**Description**	-Pre-treated with phosphoric acid<br>-Pyrolysis of hazelnut shells	-Water washed hazelnut shells<br>-Pyrolysis of hazelnut shells
**Precursor 1**	Hazelnut shells	Hazelnut shells
**Precursor 1 price range / \$ kg^{-1}**	0.15 - 0.25	
**Reference for price of precursor 1**	[6], [7], [8]	
**Precursor 2**	Phosphoric acid 85%	
**Precursor 2 price range / \$ kg^{-1}**	0.75 - 1.75	
**Reference for price**	[9]	
**Yield of production process**	16%	23%
**Production fixed cost range / \$ kg^{-1}**	2.70 - 3.30	2.25 - 2.75
**Total cost / \$ kg^{-1}**	5.65 - 9.55	2.9 - 3.85
**Average cost / \$ kg^{-1}**	7.60	3.40
**Specific capacity / mAh g^{-1}**	158	265
**Initial Coulombic efficiency**	33.69%	70.50%
**Voltage @100% SOC / V**	0.0347	0.0398
**Voltage @90% SOC / V**	0.0682	0.0716
**Voltage @80% SOC / V**	0.0868	0.0875
**Voltage @70% SOC / V**	0.1017	0.0994
**Voltage @60% SOC / V**	0.1911	0.1074
**Voltage @50% SOC / V**	0.3288	0.1352
**Voltage @40% SOC / V**	0.4777	0.175
**Voltage @30% SOC / V**	0.6489	0.2863
**Voltage @20% SOC / V**	0.8573	0.5288
**Voltage @10% SOC / V**	1.14	0.8628
**Voltage @0% SOC / V**	2	2

Table S3. Input data of NVP cathode material for cost – performance analysis.

**Code**	at-hz-HC		
**Specific capacity / mAh g^{-1}**	102		
**Total cost / \$ kg^{-1}**	25		
**Material density / g cm^{-3}**	3.2		
**Voltage vs.**	Na/Na^+	at-hz-HC	ww-hz-HC-32 µm
**Voltage @100% SOC / V**	4.2000	4.1653	4.1602
**Voltage @90% SOC / V**	3.3820	3.3138	3.3104
**Voltage @80% SOC / V**	3.3820	3.2952	3.2945
**Voltage @70% SOC / V**	3.3784	3.2767	3.2790
**Voltage @60% SOC / V**	3.3766	3.1855	3.2692
**Voltage @50% SOC / V**	3.3748	3.0460	3.2396
**Voltage @40% SOC / V**	3.3712	2.8935	3.1962
**Voltage @30% SOC / V**	3.3676	2.7187	3.0813
**Voltage @20% SOC / V**	3.3604	2.5031	2.8316
**Voltage @10% SOC / V**	3.3586	2.2186	2.4958
**Voltage @0% SOC / V**	2.500	1.3586	1.3586

The characteristics of the four types of battery packs considered in the simulations are summarized in Table S4.

Table S4. Characteristics of the simulated battery packs.

Parameter	Domestic storage battery	Grid storage battery	pHEV battery	EV battery
Number of cells per module (total)	36	4	20	20
Number of cells in parallel group in module	1	1	1	4
Number of modules in row	2	42	2	5
Number of rows of modules per pack	1	7	1	4
Number of modules in parallel	1	7	1	1
Number of packs manufactured per year	500,000	500,000	500,000	500,000
Pack energy, kWh	11.5	500	15	100
Target battery pack power at 20% SOC, kW	7	250	110	150
Power-to-energy ratio	0.61	0.5	7.3	1.50

References:

[1] P.A. Nelson, K.G. Gallagher, I.D. Bloom, D.W. Dees, Modeling the performance and cost of lithium-ion batteries for electric-drive vehicles. Argonne National Lab., Arfonne, IL (US), **2012**.

[2] L.H.B. Nguyen, P. Camacho, Sanz, J. Fondard, D. Carlier, L. Croguennec, M.R. Palacin, A. Ponrouch, C. Courrèges, R. Dedryvère, K. Trad, C. Jordy, S. Genies, Y. Reynier, L. Simonin, *J. Power Sources* **2022**, *529*, 231253.

[3] M. León, J. Silva, S. Carrasco, N. Barrientos, *Processes* **2020**, *8*, 945.

[4] G.G. Stavropoulos, A.A. Zabaniotou, *Fuel Process. Technol.* **2009**, *90*, 952–957.

[5] C. Ng, W.E. Marshall, R.M. Rao, R.R. Bansode, J.N. Losso, *Ind. Crops Prod.* **2003**, *17*, 209-217.

[6] B.E. Product, https://www.biomsrl.it/negozio/gusci/trito/, **2022**.

[7] COV Energia, http://www.cov-energia.it/, **2022**.

[8] B. Nocciola, https://www.bionocciola.it/, **2022**.

[9] ChemAnalyst, Phosphoric acid price trend and forecast, **2022**.

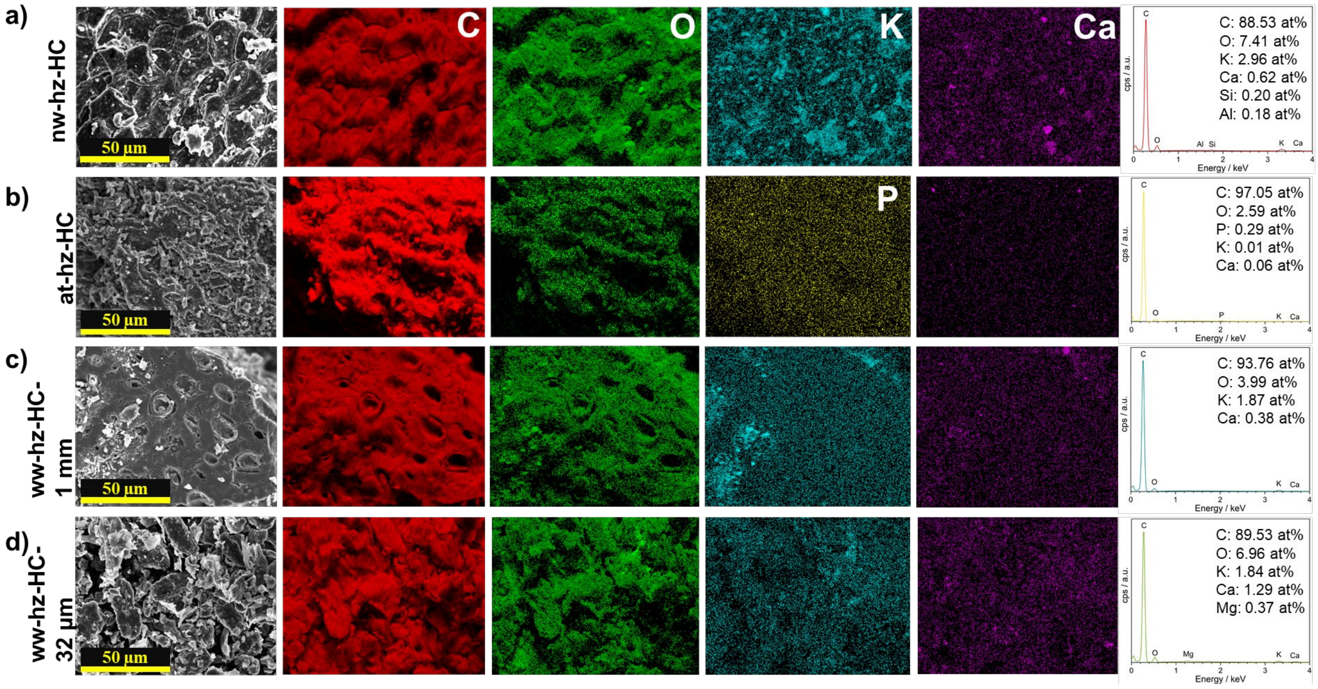

Figure S1. EDX images of the hazelnut shell-derived-HCs and atomic concentration. a) nw-hz-HC, b) at-hz-HC, c) ww-hz-HC-1 mm and d) ww-hz-HC-32 µm.

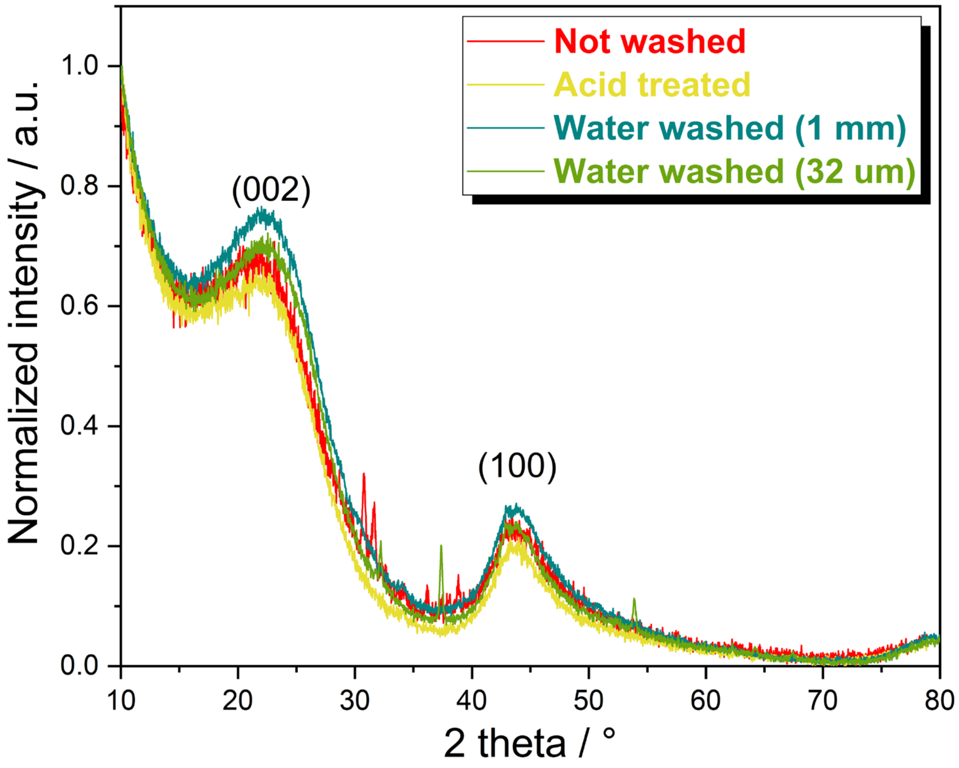

Figure S2. X-ray diffraction patterns of not washed (red line), acid-treated (yellow line), and water washed with 1 mm (dark green line) and 32 μm (light green line) particle size hazelnut-shell-derived-HC anode materials.

Table S5. (002) reflection and calculated interlayer distance (d_{002}) from XRD.

Sample	(002) peak position (2 theta/°)	d_{002} (nm)
nw-hz-HC	22.05	0.40
at-hz-HC	22.18	0.40
ww-hz-HC-1 mm	22.02	0.40
ww-hz-HC-32 μm	22.09	0.40

The (002) peak position and calculated interlayer spacing (d_{002}) from XRD indicate that the pre-treatment and particle sizes do not significantly influence the mentioned parameter. All HCs show equivalent average interlayer distance which may be due to the same pyrolysis temperature is carried out (1100 °C).

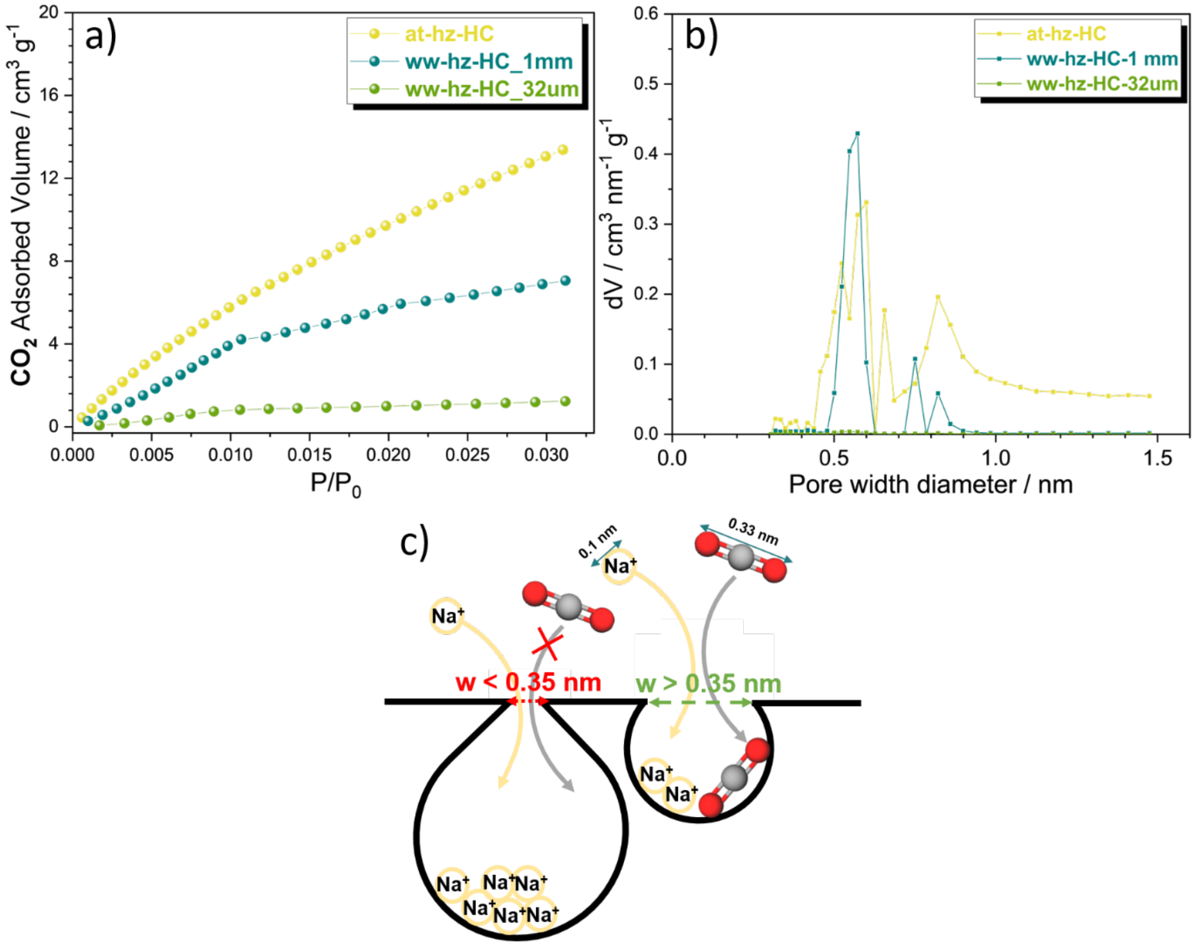

Figure S3. a) CO_2 adsorption and b) corresponding pore size distribution determined by DFT calculation of at-hz-HC, ww-hz-HC-1 mm, and ww-hz-32 µm HC. c) Scheme of pore model of ww-hz-HC-32 µm (left) and ww-hz-HC-1 mm (right), respectively.

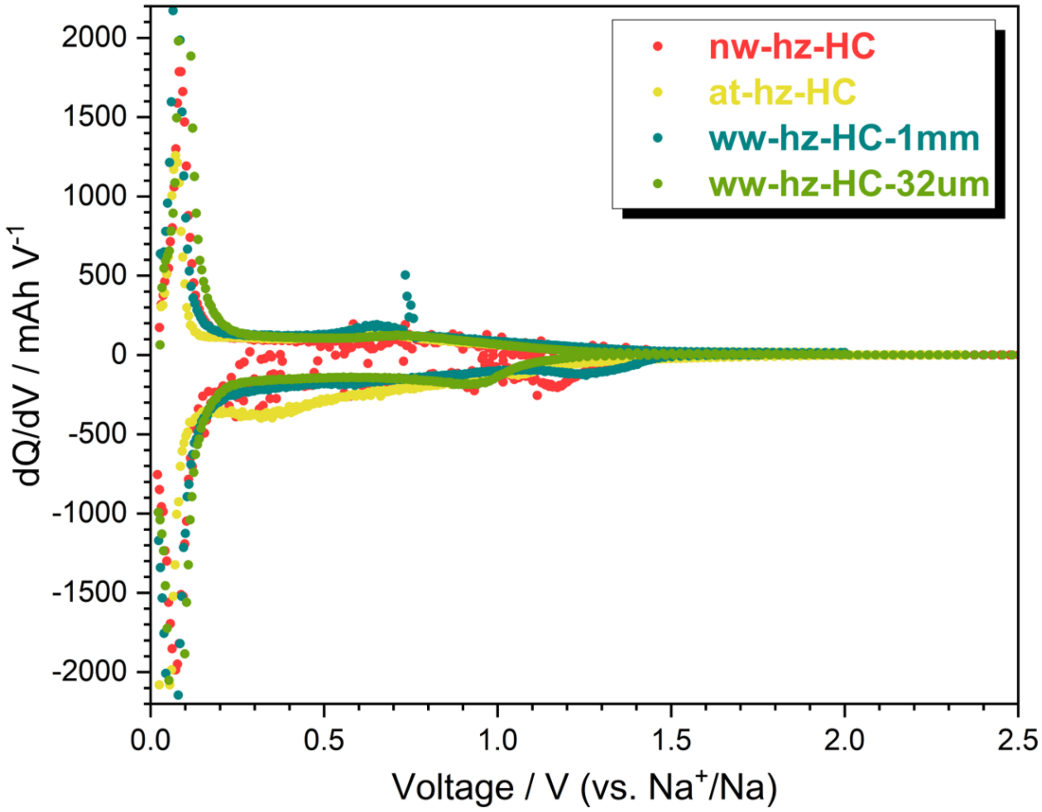

Figure S4. dQ/dV plot of the hazelnut shells-derived-HCs at the first galvanostatic cycle at 4 mA g^{-1} in half cell configuration. Counter and reference electrode Na metal, electrolyte 1 M $NaPF_6$ in EC: PC with 2 wt.% FEC solution. Active material mass loading: 2.2 mg cm^{-2}. Temperature 20 ± 2 °C.

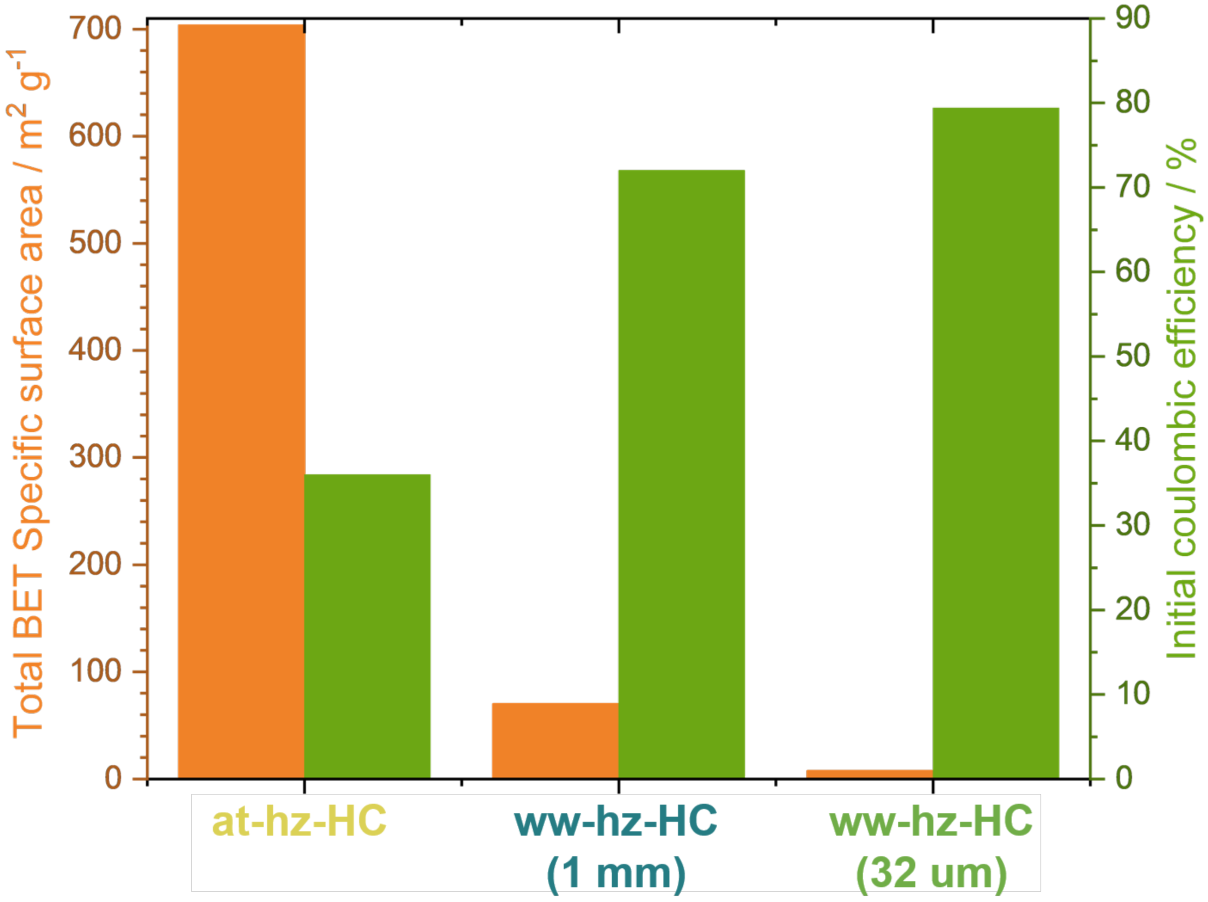

Figure S5. Correlation between the total specific surface area (SSA) and the initial Coulombic efficiency (ICE) values of the at-hz-HC, ww-hz-HC-1 mm and ww-hz-HC-32 μm hazelnut shell-derived-HCs.

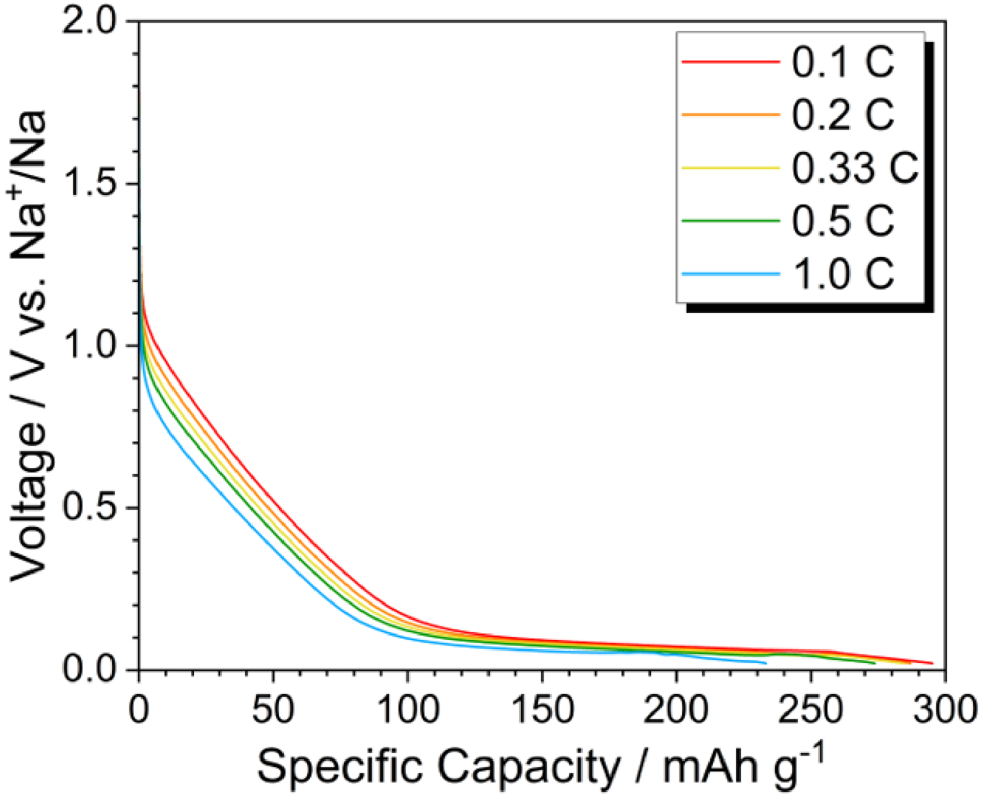

Figure S6. Voltage profiles of ww-hz-HC-32 µm in half cell at different C-rates in the voltage range of 2.0 to 0.02 V vs. Na^+/Na. Counter and reference electrode Na metal, electrolyte 1 M $NaPF_6$ in PC with 2 wt.% FEC. Temperature 20 ± 2 °C.

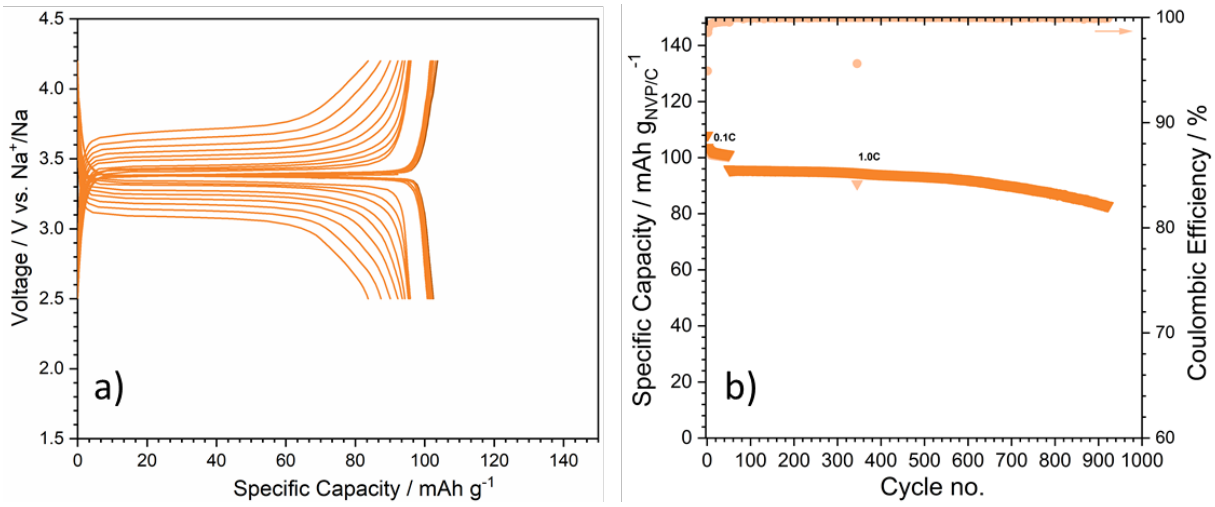

Figure S7. a) Voltage profile and b) galvanostatic cycling of NVP/C in half cell configuration in the voltage range of 4.2 to 2.5 V vs. Na^+/Na. Counter and reference electrode Na metal, electrolyte 1M $NaPF_6$ in PC with 2 wt.% FEC. Active material mass loading: 5.3 mg cm^{-2} (~5.27 mg). Temperature 20 ± 2 °C.

Table S6. Life cycle impacts analysis per kg of hazelnut shell-derived HC material processing in two synthesis routes (at-hz-HC and ww-hz-HC-32 µm) on various indicators according to ILCD 2011 midpoint+ method.

**Indicator**	**at-hz-HC**	**ww-hz-HC-32 µm**
**Acidification /molc H^+_{eq} kg_{HC}^{-1}**	12.2619	11.9079
**Climate change / kg CO_2 eq kg_{HC}^{-1}**	3462.57	3871.43
**Fresh water eco-toxicity / CTU_e kg_{HC}^{-1}**	1.02992×10^5	1.03350×10^5
**Fresh water eutrophication / kg P_{eq} kg_{HC}^{-1}**	4.55431	4.9428
**Human toxicity, cancer effects / CTU_h kg_{HC}^{-1}**	4.95096×10^{-4}	4.30474×10^{-4}
**Human toxicity, non-cancer effects / CTU_h kg_{HC}^{-1}**	1.42692×10^{-3}	1.43706×10^{-3}
**Ionising radiation E (interim) / CTU_e kg_{HC}^{-1}**	1.99682×10^{-3}	2.42263×10^{-3}
**Ionising radiation HH / kBq $U235_{eq}$ kg_{HC}^{-1}**	866.644	1043.26
**Land use / kg C deficit kg_{HC}^{-1}**	3609.4	3453.69
**Marine eutrophication / kg N_{eq} kg_{HC}^{-1}**	2.60374	2.87465
**Mineral, fossil & ren resource depletion / kg Sb_{eq} kg_{HC}^{-1}**	7.23656×10^{-2}	5.68152×10^{-2}
**Ozone depletion / kg CFC-11_{eq} kg_{HC}^{-1}**	1.007×10^{-4}	1.05408×10^{-4}
**Particulate matter / kg $PM2.5_{eq}$ kg_{HC}^{-1}**	0.85291	0.842395
**Photochemical ozone formation / kg $NMVOC_{eq}$ kg_{HC}^{-1}**	5.24857	5.60638
**Terrestrial eutrophication / molc N_{eq} kg_{HC}^{-1}**	20.2377	22.0534
**Water resource depletion / m^3 H_2O_{eq} kg_{HC}^{-1}**	3205.69	3835.02

Each indicator is calculated by allocating the corresponding environmental impacts to functional unit (*i.e.,* 1 kg of HC production). They are described in a unit of equivalence (eq) to quantify the impact caused by different material and energy flow.

Acidification

Acidification refers to the effect of corresponding emissions that have the potential to acidify soil or water agent, and they are calculated based on unit molc (moles of charge) H^+_{eq} kg_{HC}^{-1}.

Climate change

Climate change indicates the global warming potential which are caused by the emission of greenhouse gases, such as CO_2, CH_4, and N_2O, measured in kg $CO_{2\ eq}$ kg_{HC}^{-1}.

Human toxicity

Human toxicity describes the effect that can potentially cause cancer in human beings and is measured by the unit comparative toxic unit for humans (CTU_h kg_{HC}^{-1})

Resource depletion

Resource depletion means the depletion of abiotic resource, covering mineral, fossil, and renewable resource. It is measured with unit kg Sb_{eq} kg_{HC}^{-1}.

Particulate matter

Particulate matter is measured by kg $PM2.5_{eq}$ kg_{HC}^{-1} and describes the effects of fine particulates with an aerodynamic diameter of less than 2.5 µm.

Freshwater eutrophication

Freshwater eutrophication measures the impact on freshwater, which can lead to the eutrophication of water ecosystem. It is measured by kg P_{eq} kg_{HC}^{-1}.

Marine eutrophication

Freshwater eutrophication measures the impact on marine, which can lead to the eutrophication of water ecosystem. It is measured by kg N_{eq} kg_{HC}^{-1}.

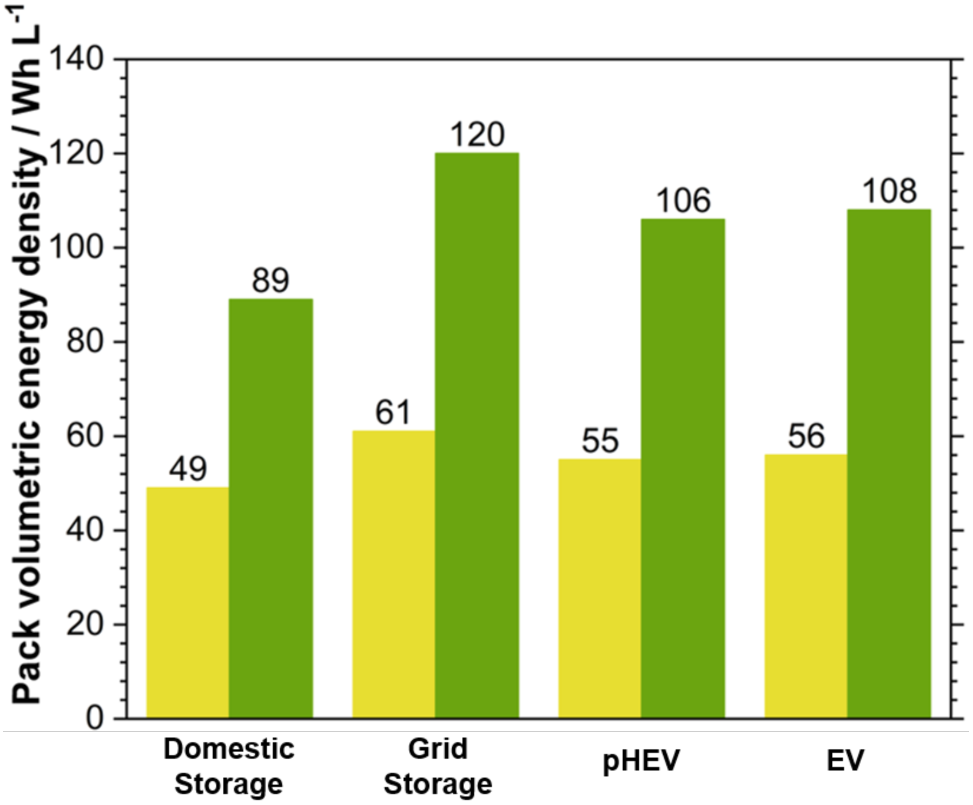

Figure S8. Analysis of the volumetric energy pack of the four SIB configurations using as an anode at-hz-HC (yellow) and ww-hz-HC-32 µm (dark green).

The simulated result using at-hz-HC or ww-hz-HC-32 um as anode in four different sodium-ion battery pack is summarized in Table S7.

Table S7. Simulated results of four different sodium-ion battery pack, *i.e.*, domestic storage, grid storage, pHEV and High-end EV using as anode at-hz-HC and ww-hz-HC-32 μm.

	**at-hz-HC**				**ww-hz-HC-32 μm**			
	**Domestic storage**	**Grid storage**	**pHEV**	**High-end EV**	**Domestic storage**	**Grid storage**	**pHEV**	**High-end EV**
**Positive active material / $/pack**	4288.30	186447.85	5593.44	37289.57	1828.88	79516.35	2385.49	15903.27
**Negative active material / $/pack**	288.96	12490.83	374.95	2505.14	68.61	2957.60	88.81	593.98
**Carbon additive / $/pack**	52.78	2294.74	68.84	458.95	22.51	978.66	29.36	195.73
**Binders / $/pack**	149.13	6482.05	194.47	1296.60	64.44	2800.00	84.00	560.15
**Solvents / $/pack**	580.19	25225.78	756.77	5045.16	247.44	10758.30	322.75	2151.66
**Positive current collector / $/pack**	74.86	3200.17	96.17	645.28	32.61	1382.97	41.60	279.94
**Negative current collector / $/pack**	77.51	3297.11	99.14	666.44	34.01	1432.03	43.10	290.91
**Separators / $/pack**	100.75	4348.87	130.56	872.81	43.36	1865.23	56.02	374.99
**Electrolyte / $/pack**	374.78	16254.13	487.75	3254.75	168.62	7302.52	219.16	1463.29
**Additives / $/pack**	0.00	0.00	0.00	0.00	0.00	0.00	0.00	0.00
**Cell purchased items / $/pack**	61.53	1314.32	49.75	359.64	49.88	879.22	36.47	265.73
**Module purchased items / $/pack**	219.43	7091.18	158.18	1019.09	202.30	5733.49	135.95	841.50
**Pack purchased items / $/pack**	324.38	6310.41	345.44	1072.08	274.72	4417.78	287.32	792.74
**Battery management system / $/pack**	222.80	666.46	422.28	383.04	221.80	664.26	393.86	367.54
**Battery system total energy / kWh**	11.45	496.71	14.93	99.61	11.44	496.23	14.91	99.47
**Battery system rated power / kW**	7.00	250.00	110.00	150.00	7.00	250.00	110.00	150.00
**Battery system capacity / Ah**	71.99	1341.47	169.03	450.73	64.00	1192.48	150.25	400.67
**Battery system nominal operating voltage / V**	159.09	370.27	88.31	220.99	178.72	416.13	99.23	248.27
**Cell volume / L**	2.19	5.51	4.89	3.33	1.05	2.58	2.30	1.58
**Cell mass / kg**	3.89	10.27	9.07	6.06	1.74	4.58	4.04	2.71
**Cell capacity / Ah**	71.99	191.64	169.03	112.68	64.00	170.35	150.25	100.17
**Module volume / L**	82.20	22.94	101.26	69.20	39.85	10.82	47.90	33.08
**Module mass / kg**	148.77	44.57	191.33	128.75	67.99	20.14	86.49	58.61
**Module capacity / Ah**	71.99	191.64	169.03	450.73	64.00	170.35	150.25	400.67
**Pack specific energy / Wh kg^{-1}**	29.43	31.46	31.46	32.66	56.45	64.15	63.21	66.35
**Pack energy density, / Wh L^{-1}**	49.08	60.72	54.86	56.48	89.18	119.99	105.79	108.24
**Pack total mass / kg**	389.20	15789.16	474.53	3049.70	202.60	7735.15	235.87	1499.17
**Pack volume / L**	233.34	8180.98	272.09	1763.48	128.25	4135.49	140.93	918.99
**Pack cost / $ $k^{-1}Wh^{-1}$**	728.75	650.54	707.79	657.84	366.22	297.15	345.35	303.43

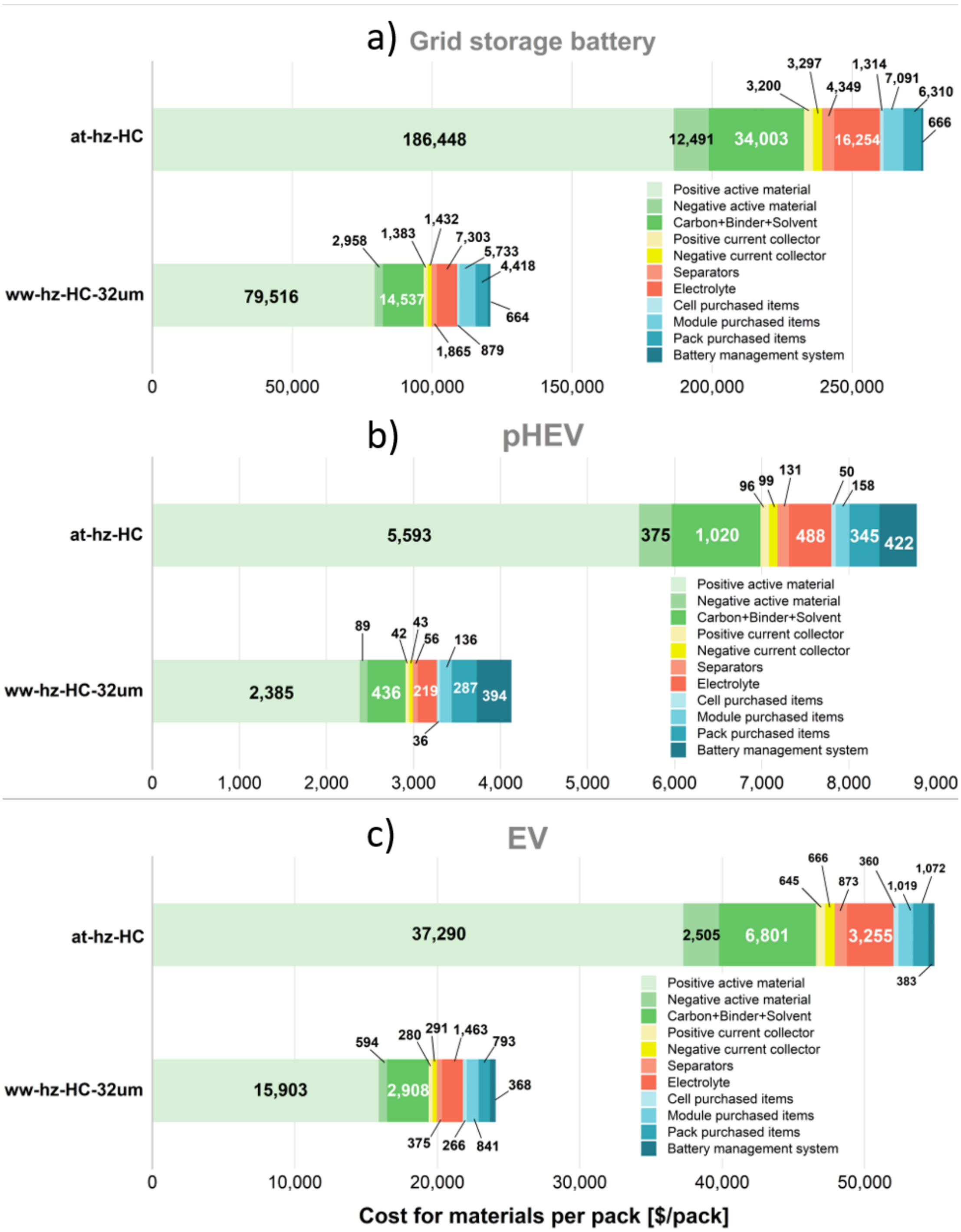

Figure S9. Comparison between at-hz-HC and ww-hz-HC-32 µm HCs of the breakdown of the material costs for a) grid energy storage battery, b) pHEV, and c) EV.
